# Supplementary material for: Vibrio parahaemolyticus from Migratory Birds in China Carries an Extra Copy of tRNA-Gly and Plasmid-Mediated Quinolone Resistance Gene qnrD
Source: Microbiol Spectr. 2023 Jun 1;11(4):e02170-22. doi: 10.1128/spectrum.02170-22 (PMC10433854; doi:10.1128/spectrum.02170-22)
Supplement: Supplemental File 1 — Tables S1 to S6. Download spectrum.02170-22-s0001.pdf, PDF file, 0.3 MB [file spectrum.02170-22-s0001.pdf]

Supplementary Table 1 The information of 124 *V. parahaemolyticus* strains in this study

Isolate name	Time	Host	Sample Type	location†	Antibiotic resistance genes	mobile elements			virulence gene	MIC value of *V. parahaemolyticus* for antibiotics																		accesion number*
						Plasmids	Insert component	Transposon		AK (μg/mL)	GM (μg/mL)	MEM (μg/mL)	IP (μg/mL)	CTX (μg/mL)	FEP (μg/mL)	CAZ (μg/mL)	ATM (μg/mL)	AC (μg/mL)	AMP (μg/mL)	AMC (μg/mL)	SAM (μg/mL)	C (μg/mL)	CIP (μg/mL)	LVX (μg/mL)	MXF (μg/mL)	TE (μg/mL)	SXT (μg/mL)	
SH2-1	2018.1	*Ardeola bacchus*	faeces	OTCWP, Shenzhen	*blaCARB-27*	none	none	none	*tlh*	16	8	0.38	0.38	2	0.25	0.125	2	1.5	8	1	1.5	1	0.38	0.25	0.5	0.19	0.19	JAQBIM000000000
SH2-2	2018.1	*Ardeola bacchus*	faeces	OTCWP, Shenzhen	*blaCARB-27*	none	none	none	*tlh*	24	3	0.064	0.125	1.5	0.25	0.19	1	12	8	1	1.5	1	0.38	0.047	0.38	0.19	0.38	JAQBIN000000000
SH4-3	2018.1	*Ardeola bacchus*	faeces	OTCWP, Shenzhen	*blaCARB-27*	none	none	none	*tlh*	12	12	0.5	0.19	0.125	0.47	0.19	2	8	2	1	0.75	1.5	0.38	0.19	0.75	0.125	0.19	JAQBIO000000000
SH5-1	2018.1	*Ardeola bacchus*	faeces	OTCWP, Shenzhen	*blaCARB-27*	none	none	none	*tlh*	16	4	0.25	0.19	0.125	0.5	0.25	1.5	12	6	1	0.75	1	0.19	0.25	0.75	0.19	0.19	JAQBIP000000000
SH7-1	2018.1	*Mallard*	faeces	OTCWP, Shenzhen	*blaCARB-25*	none	IS30,ISL3	none	*tlh*	8	3	0.125	3	0.064	0.75	0.19	2	32	32	1	3	12	0.38	0.25	0.38	0.19	0.125	JAQBIQ000000000
SH7-2	2018.1	*Mallard*	faeces	OTCWP, Shenzhen	*blaCARB-43*	none	IS3,IS5,ISL3	Tn7	*tlh*	8	6	0.094	0.19	0.094	0.38	0.064	1	4	8	1	0.75	1	0.38	0.19	0.75	0.25	0.19	JAQBIR000000000
SH8-1	2018.1	*Mallard*	faeces	OTCWP, Shenzhen	*blaCARB-25*	none	IS3,IS5,ISL3	none	*tlh*	8	3	0.016	0.25	0.38	4	0.064	2	12	32	1	1.5	0.75	0.38	0.19	0.38	0.19	0.094	JAQBIS000000000
SH8-2	2018.1	*Mallard*	faeces	OTCWP, Shenzhen	*blaCARB-35*	none	none	none	*tlh*	12	2	0.19	0.19	0.125	0.5	0.125	1.5	8	6	1	0.75	1	0.38	0.19	0.19	0.125	0.19	JAQBIT000000000
SH10-3	2018.1	*Mallard*	faeces	OTCWP, Shenzhen	*blaCARB-25, qnrD1*	pCol3M	IS5,IS256,IS3,IS4,IS481	none	*tlh*	4	1.5	0.125	0.19	0.094	0.47	0.094	2	0.75	1	0.75	1	1	0.38	0.38	0.38	0.19	0.19	JAQBIU000000000
SH10-4	2018.1	*Mallard*	faeces	OTCWP, Shenzhen	*blaCARB-29*	none	none	none	*tlh*	12	3	0.5	3	0.125	0.5	0.094	2	12	24	1	1.5	12	0.38	0.25	1.5	0.19	0.19	JAQBIV000000000
SH11-1	2018.1	*Mallard*	faeces	OTCWP, Shenzhen	*blaCARB-29*	none	none	none	*tlh*	8	2	0.016	0.125	0.047	0.5	0.064	2	0.5	1	1	0.25	1	0.38	0.125	0.25	0.125	0.125	JAQBIW000000000
SH13-1	2018.1	*Mallard*	faeces	OTCWP, Shenzhen	*blaCARB-25, qnrD1*	pCol3M	ISL3,IS3,IS481,IS5	none	*tlh*	32	2	0.25	0.19	0.094	0.5	0.064	1	3	6	1	0.75	0.75	0.38	0.125	1.5	0.19	0.19	JAQBIX000000000
SH14-1	2018.1	*Mallard*	faeces	OTCWP, Shenzhen	*blaCARB-25*	none	none	none	*tlh*	6	2	0.016	0.19	0.094	0.5	0.094	1	3	1.5	1	0.5	0.75	0.38	0.094	0.38	0.19	0.19	JAQBIY000000000
SH14-2	2018.1	*Mallard*	faeces	OTCWP, Shenzhen	*blaCARB-25, qnrD1*	pCol3M	IS630,IS5	none	*tlh*	24	2	0.25	0.19	0.094	0.75	0.125	2	1.5	2	1	0.75	12	0.19	0.25	1.5	0.19	0.25	JAQBIZ000000000
SH15-1	2018.1	*Mallard*	faeces	OTCWP, Shenzhen	*blaCARB-46*	none	none	none	*tlh*	12	3	0.016	0.19	0.125	0.75	0.25	1	8	32	1	1.5	1	0.38	0.25	0.5	0.38	0.125	JAQBJA000000000
SH15-2	2018.1	*Mallard*	faeces	OTCWP, Shenzhen	*blaCARB-25, qnrD1*	pCol3M	ISL3,IS3,IS5	none	*tlh*	16	4	0.75	0.19	0.094	0.38	0.047	1	2	4	0.19	0.75	16	0.38	0.25	1.5	0.19	1	JAQBJB000000000
SH16-1	2018.1	*Mallard*	faeces	OTCWP, Shenzhen	*blaCARB-46*	none	none	none	*tlh*	8	3	0.064	0.5	0.064	0.5	0.125	2	256	256	1	4	1	0.19	0.125	0.38	1	0.125	JAQBJC000000000
SH16-2	2018.1	*Mallard*	faeces	OTCWP, Shenzhen	*blaCARB-25, qnrD1*	pCol3M	IS481	none	*tlh*	12	3	0.19	1	0.094	0.5	0.094	2	6	8	1	4	8	0.38	0.25	1.5	0.19	0.19	JAQBJD000000000
FT2-1	2018.1	*Mallard*	faeces	FRFR, Shenzhen	*blaCARB-29*	none	none	none	*tlh*	16	3	0.016	0.19	0.125	1	0.125	1	6	12	1	1	1	0.38	0.25	0.19	0.38	0.094	JAQBIG000000000
FT2-2	2018.1	*Mallard*	faeces	FRFR, Shenzhen	*blaCARB-38*	none	none	none	*tlh*	12	3	0.016	0.19	0.064	0.5	0.094	2	0.19	0.19	1	0.25	1	0.38	0.125	0.38	0.19	0.094	JAQBIH000000000
FT3-1	2018.1	*Mallard*	faeces	FRFR, Shenzhen	*blaCARB-38*	none	none	none	*tlh*	8	3	0.032	0.19	0.064	0.38	0.25	2	24	16	1	3	8	0.19	0.125	0.38	1.5	0.094	JAQBII000000000
FT3-2	2018.1	*Mallard*	faeces	FRFR, Shenzhen	*blaCARB-31*	none	none	none	*tlh*	16	3	0.047	0.38	0.064	0.75	0.25	1	32	256	1	3	8	0.38	0.125	0.5	1	0.125	JAQBIJ000000000
FT8-1	2018.1	*Ardeola bacchus*	faeces	FRFR, Shenzhen	*blaCARB-35*	none	none	none	*tlh*	16	3	0.047	0.125	0.094	0.75	0.125	1	1.5	2	1	0.75	0.75	0.19	0.75	0.38	0.19	0.094	JAQBIK000000000
FT8-2	2018.1	*Ardeola bacchus*	faeces	FRFR, Shenzhen	*blaCARB-43*	none	none	none	*tlh*	8	3	0.19	0.19	0.094	0.5	0.094	1	6	6	1	0.75	1	0.38	0.19	0.38	0.19	0.094	JAQBIL000000000
1-1	2019.3	*Charadriiformes*	faeces	OTCWP, Shenzhen	*blaCARB-26*	none	none	none	*tlh*	32	6	0.016	0.19	1.5	1	8	2	8	12	1.5	1	1	0.38	0.125	0.38	0.19	0.094	JAQBJE000000000
1-2	2019.3	*Charadriiformes*	faeces	OTCWP, Shenzhen	*blaCARB-33*	none	IS110，IS1595，IS200_IS605,IS21,IS256,IS3,IS481,IS5,ISL3	Tn7	*tlh*	16	2	0.016	0.19	0.047	0.25	0.19	2	16	12	2	1	1	0.016	0.094	0.5	0.125	0.094	JAQBJF000000000
2-1	2019.3	*Charadriiformes*	faeces	OTCWP, Shenzhen	*blaCARB-33*	none	IS110,IS200_IS605,IS256,IS3,IS4,IS481,ISL3	none	*tlh*	16	6	0.016	0.38	2	0.38	8	1.5	12	12	1	1	1	0.125	0.094	0.5	0.125	0.19	JAQBJG000000000
3-1	2019.3	*Mallard*	faeces	OTCWP, Shenzhen	*blaCARB-47*	none	IS3,IS5,ISL3	none	*tlh*	32	8	0.016	0.19	0.125	0.25	0.125	1.5	4	8	1	1	1.5	0.125	0.125	0.38	0.38	0.19	JAQBJH000000000
3-2	2019.3	*Mallard*	faeces	OTCWP, Shenzhen	*blaCARB-31, blaCARB-44*	none	none	none	*tlh*	6	3	0.016	0.19	0.094	0.25	0.094	1.5	24	32	1.5	1	0.75	0.25	0.25	0.19	0.125	0.125	JAQBJI000000000
4-1	2019.3	*Charadriiformes*	faeces	OTCWP, Shenzhen	*blaCARB-22, blaCARB-35, blaCARB-38, blaCARB-47, qnrC*	none	ISL3,IS110,IS3,IS5	none	*tlh*	6	1.5	0.016	0.19	0.094	0.75	0.094	1	6	32	1.5	1	0.75	0.125	0.25	0.38	0.19	0.125	JAQBJJ000000000
4-2	2019.3	*Charadriiformes*	faeces	OTCWP, Shenzhen	*blaCARB-22, blaCARB-35, blaCARB-38, blaCARB-47, qnrC*	none	ISL3,IS110,IS3,IS5	none	*tlh*	6	3	0.016	0.19	0.094	0.5	0.125	1	4	8	2	0.75	1	0.016	0.25	0.25	0.19	0.094	JAQBJK000000000
5-1	2019.3	*Mallard*	faeces	OTCWP, Shenzhen	*blaCARB-46*	none	none	none	*tlh*	16	6	0.016	0.19	0.125	0.75	0.25	1.5	6	32	1	1	1	0.38	0.19	0.38	0.19	0.094	JAQBJL000000000
5-2	2019.3	*Mallard*	faeces	OTCWP, Shenzhen	*blaCARB-38*	none	none	none	*tlh*	16	6	0.016	0.19	0.064	0.25	0.064	2	24	32	1	0.75	0.75	0.38	0.016	0.38	0.19	0.19	JAQBJM000000000
6-1	2019.3	*Mallard*	faeces	OTCWP, Shenzhen	*blaCARB-33*	none	IS110,IS1595,IS200_IS605,IS21,IS256,IS3,IS481,IS5,ISL3	Tn7	*tlh*	32	8	0.016	0.19	0.125	0.25	0.125	2	24	8	1	1	1.5	0.19	0.19	0.38	0.19	0.125	JAQBJN000000000
6-2	2019.3	*Mallard*	faeces	OTCWP, Shenzhen	*blaCARB-33*	none	none	none	*tlh*	6	3	0.016	0.19	1.5	0.25	0.19	1.5	1	16	1.5	0.75	1	0.38	0.25	0.19	0.19	0.125	JAQBJO000000000
7-1	2019.3	*Mallard*	faeces	OTCWP, Shenzhen	*blaCARB-48*	none	none	none	*tlh*	12	4	0.016	0.19	0.094	0.25	0.094	1.5	24	32	2	1.5	1	0.38	0.19	0.75	0.38	0.125	JAQBJP000000000
7-2	2019.3	*Mallard*	faeces	OTCWP, Shenzhen	*blaCARB-24*	none	IS3,IS481,IS66	none	*tlh*	8	1.5	0.016	0.38	0.047	0.38	0.19	1	8	32	1	1	1	0.38	0.047	0.5	0.125	0.125	JAQBJQ000000000
8-1	2019.3	*Mallard*	faeces	OTCWP, Shenzhen	*blaCARB-29, qnrC*	none	ISL3,IS110,IS200_IS605,IS21,IS3,IS630	none	*tlh*	6	8	0.016	0.38	1.5	0.38	0.25	1.5	8	8	1	1	0.75	0.19	0.19	0.38	0.125	0.125	JAQBJR000000000
8-2	2019.3	*Mallard*	faeces	OTCWP, Shenzhen	*blaCARB-25, blaCARB-35, blaCARB-47*	none	IS1595,IS3,IS30,ISNCY	none	*tlh*	12	1.5	0.016	0.19	0.38	0.75	8	1	8	24	2	1.5	1	0.38	0.25	0.75	0.19	0.125	JAQBJS000000000
9-1	2019.3	*Charadriiformes*	faeces	OTCWP, Shenzhen	*blaCARB-29, blaCARB-33, blaCARB-41*	none	none	none	*tlh*	6	3	0.016	0.19	1.5	2	8	2	24	24	1.5	1.5	1.5	0.19	0.25	0.25	0.19	0.125	JAQBJT000000000
9-2	2019.3	*Charadriiformes*	faeces	OTCWP, Shenzhen	*blaCARB-29, blaCARB-33, blaCARB-41*	none	none	none	*tlh*	6	1.5	0.016	0.19	2	1	8	3	32	32	1	1.5	0.75	0.125	0.25	0.38	0.125	0.19	JAQBJU000000000
10-1	2019.3	*Charadriiformes*	faeces	OTCWP, Shenzhen	*blaCARB-44, blaCARB-46*	none	none	none	*tlh*	16	3	0.016	0.19	0.094	0.094	1	2	16	16	1	1	1	0.38	0.19	0.5	0.125	0.125	JAQBJV000000000
10-2	2019.3	*Charadriiformes*	faeces	OTCWP, Shenzhen	*blaCARB-44, blaCARB-46*	none	none	none	*tlh*	6	2	0.016	0.38	2	0.75	0.19	2	6	8	1	1	1	0.094	0.25	0.38	0.38	0.125	JAQBJW000000000
11-1	2019.3	*Charadriiformes*	faeces	OTCWP, Shenzhen	*blaCARB-24*	none	none	none	*tlh*	24	12	0.016	0.125	0.094	0.047	0.094	2	12	24	2	1.5	1	0.016	0.25	0.5	0.19	0.125	JAQBJX000000000
11-2	2019.3	*Charadriiformes*	faeces	OTCWP, Shenzhen	*blaCARB-33*	none	none	none	*tlh*	8	3	0.016	0.19	0.094	0.38	0.064	2	24	32	1	2	0.75	0.38	0.25	0.38	0.125	0.19	JAQBJY000000000
12-1	2019.3	*Charadriiformes*	faeces	OTCWP, Shenzhen	*blaCARB-26*	none	IS256,IS3,IS630	none	*tlh*	24	8	0.016	0.19	0.125	0.25	0.25	1.5	6	32	2	0.75	1	0.38	0.19	0.5	0.19	0.125	JAQBJZ000000000
13-1	2019.3	*Mallard*	faeces	OTCWP, Shenzhen	*blaCARB-33*	none	none	none	*tlh*	6	2	0.016	0.19	0.094	0.25	0.19	1	6	48	1	1.5	1	0.125	0.125	0.75	0.19	0.19	JAQBKA000000000
13-2	2019.3	*Mallard*	faeces	OTCWP, Shenzhen	*blaCARB-21, blaCARB-30*	none	none	none	*tlh*	6	1	0.016	0.19	0.125	0.25	0.125	1.5	8	32	1	1	1	0.38	0.38	0.5	0.19	0.094	JAQBKB000000000
14-1	2019.3	*Charadriiformes*	faeces	OTCWP, Shenzhen	*blaCARB-33*	none	none	none	*tlh*	6	6	0.016	0.38	1.5	0.75	1	2	8	32	1	1.5	1	0.016	0.125	0.25	0.125	0.125	JAQBKC000000000
14-2	2019.3	*Charadriiformes*	faeces	OTCWP, Shenzhen	*blaCARB-33, blaCARB-41*	none	IS1595,IS256,IS4,IS481,IS5,IS630	none	*tlh*	24	8	0.016	0.19	0.094	0.25	0.125	1	0.25	6	1	0.25	1	0.38	0.25	0.5	0.125	0.125	JAQBKD000000000
15-1	2019.3	*Mallard*	faeces	OTCWP, Shenzhen	*blaCARB-33, dfrA6*	none	ISNCY,IS1595,IS256,IS3,IS30,IS5,IS630	none	*tlh*	24	8	0.016	0.38	0.125	0.25	0.125	1	6	12	1	0.75	0.75	0.9	0.25	0.5	0.125	0.19	JAQBKE000000000
15-2	2019.3	*Mallard*	faeces	OTCWP, Shenzhen	*blaCARB-33*	none	none	none	*tlh*	8	2	0.016	0.19	0.094	0.25	0.094	1	6	2	1	1.5	1	0.38	0.25	0.38	0.19	0.125	JAQBKF000000000
16-1	2019.3	*Charadriiformes*	faeces	OTCWP, Shenzhen	*blaCARB-17, blaCARB-20*	none	IS3,IS5,ISL3	none	*tlh*	6	1	0.016	0.19	1.5	0.75	0.094	1	6	32	1	1	1	0.38	0.25	0.38	0.19	0.125	JAQBKG000000000
16-2	2019.3	*Charadriiformes*	faeces	OTCWP, Shenzhen	*blaCARB-24*	none	IS3,IS5	none	*tlh*	24	8	0.016	0.19	0.094	0.125	0.094	1.5	6	12	1	1.5	1	0.38	0.25	0.38	0.19	0.125	JAQBKH000000000
17-1	2019.3	*Charadriiformes*	faeces	OTCWP, Shenzhen	*blaCARB-33*	none	none	none	*tlh*	16	3	0.016	0.19	0.094	0.75	0.094	1	2	8	1	1	1	0.38	0.25	0.38	0.19	0.125	JAQBKI000000000
18-1	2019.3	*Charadriiformes*	faeces	OTCWP, Shenzhen	*blaCARB-17, blaCARB-20*	none	ISL3,IS3,IS5	none	*tlh*	8	6	0.016	0.19	0.094	0.75	0.064	2	6	32	1	0.75	1	0.38	0.25	0.38	0.19	0.19	JAQBKJ000000000
18-2	2019.3	*Charadriiformes*	faeces	OTCWP, Shenzhen	*blaCARB-25, blaCARB-29, blaCARB-33, blaCARB-41*	none	none	none	*tlh*	24	8	0.016	0.19	0.047	0.75	0.064	1	12	32	2	1	0.75	0.38	0.19	0.38	0.38	0.19	JAQBKK000000000
20-1	2019.3	*Charadriiformes*	faeces	OTCWP, Shenzhen	*blaCARB-35, blaCARB-48*	none	none	none	*tlh*	6	3	0.016	0.38	0.094	0.25	0.125	0.5	12	8	1	1.5	1	0.016	0.38	0.5	0.125	0.094	JAQBKL000000000
20-2	2019.3	*Charadriiformes*	faeces	OTCWP, Shenzhen	*blaCARB-24*	none	none	none	*tlh*	24	8	0.016	0.19	0.064	0.75	0.094	1	12	32	2	1.5	0.75	0.38	0.25	0.5	0.125	0.125	JAQBKM000000000
21-1	2019.3	*Charadriiformes*	faeces	OTCWP, Shenzhen	*blaCARB-19*	none	IS481	none	*tlh*	24	8	0.016	0.19	0.094	0.75	0.094	2	3	32	0.5	0.38	1	0.38	0.25	0.19	0.19	0.19	JAQBKN000000000
21-2	2019.3	*Charadriiformes*	faeces	OTCWP, Shenzhen	*blaCARB-18, blaCARB-33, blaCARB-45*	none	none	none	*tlh*	6	8	0.016	0.19	0.094	0.25	0.125	2	4	8	1	0.75	0.75	0.38	0.38	0.38	0.19	0.19	JAQBKO000000000
23-1	2019.3	*Charadriiformes*	faeces	OTCWP, Shenzhen	*blaCARB-47*	none	none	none	*tlh*	6	3	0.016	0.19	0.094	0.25	0.25	1	6	12	0.5	1.5	1	0.38	0.25	0.25	0.19	0.125	JAQBKP000000000
23-2	2019.3	*Charadriiformes*	faeces	OTCWP, Shenzhen	*blaCARB-26*	none	none	none	*tlh*	6	8	0.016	0.19	0.094	0.25	0.064	1	12	32	1	1.5	0.75	0.38	0.25	0.5	0.125	0.19	JAQBKQ000000000
24-1	2019.3	*Charadriiformes*	faeces	OTCWP, Shenzhen	*blaCARB-33*	none	none	none	*tlh*	6	8	0.016	0.19	0.064	0.25	0.094	1	3	6	1	1.5	1	0.38	0.25	0.38	0.19	0.125	JAQBKR000000000
24-2	2019.3	*Charadriiformes*	faeces	OTCWP, Shenzhen	*blaCARB-20*	none	none	none	*tlh*	16	6	0.016	0.19	2	0.5	8	1	8	32	0.5	2	1	0.38	0.38	0.38	0.125	0.19	JAQBKS000000000
25-1	2019.3	*Charadriiformes*	faeces	OTCWP, Shenzhen	*blaCARB-21*	none	none	none	*tlh*	6	3	0.016	0.19	0.064	0.125	0.094	1	3	6	1	1	1	0.38	0.25	0.38	0.25	0.125	JAQBKT000000000
25-2	2019.3	*Charadriiformes*	faeces	OTCWP, Shenzhen	*blaCARB-24*	none	IS3,IS481,IS630.ISL3	Tn7	*tlh*	16	6	0.016	0.19	2	0.75	8	1	1	8	1	1	1	0.38	0.25	0.38	0.19	0.125	JAQBKU000000000
26-1	2019.3	*Mallard*	faeces	OTCWP, Shenzhen	*blaCARB-17, blaCARB-20*	none	ISL3,IS3,IS5	none	*tlh*	8	3	0.016	0.19	0.094	0.25	0.094	1	6	32	1	1	1	0.38	0.38	0.38	0.125	0.25	JAQBKV000000000
26-2	2019.3	*Mallard*	faeces	OTCWP, Shenzhen	*blaCARB-33*	none	none	none	*tlh*	16	3	0.016	0.19	0.094	0.75	0.094	1	4	8	1	1	1	0.19	0.25	0.19	0.125	0.125	JAQBKW000000000
27-1	2019.3	*Charadriiformes*	faeces	OTCWP, Shenzhen	*blaCARB-33*	none	IS110,IS200_IS605,IS21,IS3,IS481,IS5,ISL3	Tn7	*tlh*	16	2	0.016	0.19	0.094	0.25	0.094	1	6	12	1	1	1	0.38	0.25	0.38	0.25	0.19	JAQBKX000000000
27-2	2019.3	*Charadriiformes*	faeces	OTCWP, Shenzhen	*blaCARB-43*	none	none	none	*tlh*	6	2	0.016	0.19	0.094	0.125	0.064	1	2	6	0.5	1	0.75	0.38	0.25	0.19	0.19	0.125	JAQBKY000000000
28-1	2019.3	*Mallard*	faeces	OTCWP, Shenzhen	*blaCARB-33*	none	IS110,IS200_IS605,IS3,IS5,IS630,ISL3	Tn7	*tlh*	16	6	0.016	0.19	0.094	0.25	0.047	1	1.5	6	0.25	1	1	0.38	0.25	0.38	0.19	1	JAQBKZ000000000
28-2	2019.3	*Mallard*	faeces	OTCWP, Shenzhen	*blaCARB-24*	none	none	none	*tlh*	24	6	0.016	0.19	0.094	0.125	0.094	1	6	12	1	1	1	0.38	0.25	0.19	0.19	0.125	JAQBLA000000000
29-1	2019.3	*Mallard*	faeces	OTCWP, Shenzhen	*blaCARB-35*	none	none	none	*tlh*	8	8	0.016	0.19	0.094	0.75	0.047	1	2	8	1	0.5	1	0.38	0.25	0.38	0.125	0.19	JAQBLB000000000
29-2	2019.3	*Mallard*	faeces	OTCWP, Shenzhen	*blaCARB-24, qnrC*	none	IS5,IS110,IS200_IS605,IS3,IS481	none	*tlh*	24	12	0.016	0.19	0.094	0.75	0.094	1	2	32	0.25	0.5	1	0.38	0.25	0.38	0.19	0.125	JAQBLC000000000
31-1	2019.3	*Charadriiformes*	faeces	OTCWP, Shenzhen	*blaCARB-25, blaCARB-35*	none	none	none	*tlh*	6	2	0.016	0.19	0.064	0.25	0.047	0.5	2	6	1	1	1	0.19	0.5	0.19	0.19	0.25	JAQBLD000000000
31-2	2019.3	*Charadriiformes*	faeces	OTCWP, Shenzhen	*blaCARB-22, blaCARB-38*	none	ISL3	none	*tlh*	16	8	0.016	0.19	0.094	0.38	0.094	1	2	32	1	0.25	0.75	0.38	0.25	0.38	0.19	0.19	JAQBLE000000000
32-1	2019.3	*Mallard*	faeces	OTCWP, Shenzhen	*blaCARB-25, blaCARB-35*	none	none	none	*tlh*	16	2	0.016	0.38	0.125	0.25	0.094	1	2	6	0.5	1	1	0.19	0.125	0.38	0.38	0.125	JAQBLF000000000
32-2	2019.3	*Mallard*	faeces	OTCWP, Shenzhen	*blaCARB-25, blaCARB-35*	none	none	none	*tlh*	6	3	0.016	0.19	0.094	0.5	0.125	1	2	24	1	0.5	0.25 S	0.25	0.125	0.19	0.19	0.25	JAQBLG000000000
33-1	2019.3	*Charadriiformes*	faeces	OTCWP, Shenzhen	*blaCARB-33*	none	IS110,IS200_IS605,IS3,IS5,IS630,ISL3	none	*tlh*	24	12	0.016	0.19	0.125	0.25	0.094	1	4	6	1.5	1.5	1	0.38	0.25	0.19	0.19	0.125	JAQBLH000000000
33-2	2019.3	*Charadriiformes*	faeces	OTCWP, Shenzhen	*blaCARB-33*	none	IS110,IS200_IS605,IS3,IS5,IS630,ISL3	none	*tlh*	6	3	0.016	0.19	0.094	0.25	0.047	0.5	3	4	1	0.75	1	0.38	0.125	0.19	1	0.125	JAQBLI000000000
34-1	2019.3	*Charadriiformes*	faeces	OTCWP, Shenzhen	*fos*	none	ISL3,IS30,IS481,IS5	none	*tlh*	6	12	0.016	0.19	0.125	0.25	0.094	2	0.38	0.75	1	0.5	1	0.38	0.25	0.38	0.19	0.125	JAQBLJ000000000
34-2	2019.3	*Charadriiformes*	faeces	OTCWP, Shenzhen	*blaCARB-31, blaCARB-44*	none	none	none	*tlh*	24	8	0.016	0.19	0.125	0.25	0.094	2	2	8	0.25	1	1	0.38	0.25	0.19	0.38	0.25	JAQBLK000000000
35-1	2019.3	*Charadriiformes*	faeces	OTCWP, Shenzhen	*blaCARB-44*	none	none	none	*tlh*	6	2	0.016	0.19	0.094	0.5	0.047	1	8	32	0.5	2	1	0.38	0.25	0.19	0.19	0.25	JAQBLL000000000
35-2	2019.3	*Charadriiformes*	faeces	OTCWP, Shenzhen	*blaCARB-44*	none	none	none	*tlh*	16	3	0.016	0.19	0.125	0.75	0.094	1	6	6	1	0.25	1	0.19	0.25	0.38	0.19	0.125	JAQBLM000000000
36-1	2019.3	*Mallard*	faeces	OTCWP, Shenzhen	*blaCARB-28*	none	none	none	*tlh*	16	2	0.016	0.19	0.125	0.5	0.094	1	8	12	0.5	1.5	1	0.38	0.25	0.38	0.19	0.125	JAQBLN000000000
36-2	2019.3	*Mallard*	faeces	OTCWP, Shenzhen	*blaCARB-33*	none	none	none	*tlh*	24	8	0.016	0.19	0.125	0.75	0.094	1	4	8	1	2	0.75	0.38	0.125	0.38	0.19	0.125	JAQBLO000000000
37-1	2019.3	*Mallard*	faeces	OTCWP, Shenzhen	*blaCARB-46*	none	none	none	*tlh*	8	12	0.016	0.38	0.125	0.5	0.094	1	12	24	1	2	1	0.19	0.25	0.38	0.38	0.125	JAQBLP000000000
37-2	2019.3	*Mallard*	faeces	OTCWP, Shenzhen	*blaCARB-28*	none	none	none	*tlh*	8	3	0.016	0.19	0.125	0.5	0.094	1	12	16	1	2	1	0.38	0.125	0.38	0.19	0.125	JAQBLQ000000000
38-1	2019.3	*Mallard*	faeces	OTCWP, Shenzhen	*blaCARB-47*	none	IS1595,IS3,IS30,IS481,ISAs1,ISL3	none	*tlh*	24	8	0.016	0.19	0.125	0.5	0.125	1	3	12	2	1	0.75	0.38	0.25	0.19	0.19	0.25	JAQBLR000000000
38-2	2019.3	*Mallard*	faeces	OTCWP, Shenzhen	*blaCARB-29, blaCARB-33, blaCARB-41*	none	none	none	*tlh*	16	2	0.016	0.19	0.094	0.75	0.094	1	8	8	1	1	1	0.38	0.5	0.38	0.25	0.125	JAQBLS000000000
39-1	2019.3	*Mallard*	faeces	OTCWP, Shenzhen	*blaCARB-31, blaCARB-44*	none	IS1595,IS200_IS605,IS256,IS4,IS481,ISL3	none	*tlh*	8	2	0.016	0.19	0.094	0.75	0.047	1	48	48	4	1	1	0.38	0.25	0.19	0.19	0.25	JAQBLT000000000
40-1	2019.3	*Charadriiformes*	faeces	OTCWP, Shenzhen	*blaCARB-44*	none	none	none	*tlh*	6	2	0.016	0.38	0.094	0.75	0.125	1	8	32	1.5	1.5	1	0.38	0.25	0.38	0.19	0.19	JAQBLU000000000
40-2	2019.3	*Charadriiformes*	faeces	OTCWP, Shenzhen	*blaCARB-28*	none	none	none	*tlh*	16	2	0.016	0.19	0.094	0.75	0.094	1.5	8	32	2	1	1	0.38	0.25	0.38	0.19	0.25	JAQBLV000000000
41-1	2019.3	*Mallard*	faeces	OTCWP, Shenzhen	*blaCARB-33*	none	none	none	*tlh*	16	8	0.016	0.19	0.094	0.75	0.094	1	16	48	3	1.5	1	0.38	0.25	0.38	0.38	0.19	JAQBLW000000000
41-2	2019.3	*Mallard*	faeces	OTCWP, Shenzhen	*blaCARB-28*	none	none	none	*tlh*	8	2	0.016	0.19	0.047	0.25	0.125	1	32	24	1	1.5	1	0.38	0.19	0.38	0.19	0.19	JAQBLX000000000
42-1	2019.3	*Mallard*	faeces	OTCWP, Shenzhen	*blaCARB-33*	none	IS110,IS200_IS605,IS21,IS3,IS481,IS5,ISL3	Tn7	*tlh*	8	3	0.016	0.38	0.094	0.75	0.25	1.5	24	32	1	2	0.75	0.38	0.25	0.38	1	0.125	JAQBLY000000000
42-2	2019.3	*Mallard*	faeces	OTCWP, Shenzhen	*blaCARB-29, blaCARB-33, blaCARB-41*	none	none	none	*tlh*	8	2	0.016	0.19	0.25	0.25	0.047	1	4	8	0.5	2	1	0.38	0.25	0.38	0.19	0.125	JAQBLZ000000000
WS1-1	2019.3	*Water*	water	OTCWP, Shenzhen	*blaCARB-29, blaCARB-33, blaCARB-41*	none	none	none	*tlh*	6	3	0.016	0.19	0.094	0.25	0.094	1	4	8	1.5	1	1	0.38	0.25	0.19	0.75	0.25	JAQBMR000000000
F7-1	2019.3	*Charadriiformes*	faeces	FRFR, Shenzhen	*blaCARB-29, blaCARB-33, blaCARB-41*	none	none	none	*tlh*	8	2	0.016	0.19	0.094	0.25	0.047	1	6	32	1	2	1	0.38	0.25	0.38	0.19	0.19	JAQBMA000000000
F7-2	2019.3	*Charadriiformes*	faeces	FRFR, Shenzhen	*blaCARB-26*	none	IS3,IS481,IS66	none	*tlh*	16	6	0.016	0.19	0.094	0.75	0.094	2	3	32	1	2	1	0.38	0.19	0.38	0.19	0.19	JAQBMB000000000
F14-1	2019.3	*Charadriiformes*	faeces	FRFR, Shenzhen	*blaCARB-29, blaCARB-33, blaCARB-41*	none	none	none	*tlh*	24	8	0.016	0.19	0.25	0.5	0.125	1	6	6	2	1	1	0.38	0.25	0.38	0.19	0.125	JAQBMC000000000
F14-2	2019.3	*Charadriiformes*	faeces	FRFR, Shenzhen	*blaCARB-29, blaCARB-33, blaCARB-41*	none	none	none	*tlh*	6	2	0.016	0.19	0.094	0.75	0.094	2	8	32	1	2	1	0.19	0.25	0.38	0.25	0.125	JAQBMD000000000
WF2-1	2019.3	*Water*	water	FRFR, Shenzhen	*blaCARB-45*	none	none	none	*tlh*	16	3	0.016	0.19	0.094	0.75	0.125	1	6	32	2	1	1	0.38	0.25	0.38	0.19	0.19	JAQBMP000000000
WF2-2	2019.3	*Water*	water	FRFR, Shenzhen	*blaCARB-33*	none	none	none	*tlh*	8	2	0.016	0.19	2	4	4	1	4	16	1	3	1	0.38	0.125	0.38	0.19	0.19	JAQBMQ000000000
Z8-1	2019.3	*Ardeola bacchus*	faeces	Zhanjiang	*blaCARB-31*	none	IS5,ISL3	none	*tlh*	16	4	0.016	0.19	2	4	4	1	4	12	1	2	1	0.38	0.25	0.38	0.19	0.19	JAQBMS000000000
Z11-1	2019.3	*Ardeola bacchus*	faeces	Zhanjiang	*blaCARB-31, blaCARB-44, blaCARB-46, fos, qnrC*	none	IS5,ISL3	none	*tlh*	8	3	0.016	0.19	2	4	4	2	8	32	1	2	1	0.38	0.25	0.19	0.19	0.125	JAQBMT000000000
Z11-2	2019.3	*Ardeola bacchus*	faeces	Zhanjiang	*blaCARB-25, blaCARB-29, blaCARB-33, blaCARB-41*	none	ISL3,IS110,IS3	Tn7	*tlh*	8	3	0.016	0.19	0.094	0.75	0.094	1	32	24	1	2	1	0.38	0.25	0.38	0.25	0.19	JAQBMU000000000
Z13-1	2019.3	*Ardeola bacchus*	faeces	Zhanjiang	*blaCARB-46*	none	IS110,IS21,IS256,IS3,IS481,IS5,IS630,IS66,ISL3	Tn7	*tlh*	8	6	0.016	0.19	0.125	0.5	0.094	0.25	2	32	0.5	2	1	0.38	0.25	0.38	1	0.19	JAQBMV000000000
Z13-2	2019.3	*Ardeola bacchus*	faeces	Zhanjiang	*blaCARB-46*	none	IS110,IS21,IS256,IS3,IS481,IS5,IS630,IS66,ISL3	Tn7	*tlh*	6	2	0.016	0.19	0.125	0.5	0.094	1	2	32	1	3	1	0.38	0.25	0.38	0.25	0.19	JAQBMW000000000
Z14-1	2019.3	*Ardeola bacchus*	faeces	Zhanjiang	*blaCARB-35, blaCARB-43*	none	none	none	*tlh*	24	8	0.016	0.19	0.094	0.5	0.125	1	8	32	1	3	1	0.38	0.25	0.38	0.19	0.125	JAQBMX000000000

Isolate name	Time	Host	Sample Type	location†	Antibiotic resistance genes	mobile elements			virulence gene	MIC value of *V. parahaemolyticus* for antibiotics																		accesion number*
						Plasmids	Insert component	Transposon		AK (μg/mL）	GM (μg/mL）	MEM (μg/mL）	IP (μg/mL）	CTX (μg/mL）	FEP (μg/mL）	CAZ (μg/mL）	ATM (μg/mL）	AC (μg/mL）	AMP (μg/mL）	AMC (μg/mL）	SAM (μg/mL）	C (μg/mL）	CIP (μg/mL）	LVX (μg/mL）	MXF (μg/mL）	TE (μg/mL）	SXT (μg/mL）	
Z14-2	2019.3	*Ardeola bacchus*	faeces	Zhanjiang	*blaCARB-46*	none	IS110,IS21,IS256,IS3,IS481,IS5,IS630,IS66,ISL3	Tn7	*tlh*	16	8	0.016	0.19	0.094	0.5	0.094	1	4	12	1	2	1	0.38	0.25	0.19	0.19	0.125	JAQBMY000000000
Z19-1	2019.3	*Ardeola bacchus*	faeces	Zhanjiang	*blaCARB-46*	none	IS110,IS21,IS256,IS3,IS481,IS5,IS630,IS66,ISL3	Tn7	*tlh*	6	3	0.016	0.19	0.094	0.5	0.094	2	3	8	0.5	2	1	0.38	0.25	0.19	0.19	0.125	JAQBMZ000000000
L2-1	2019.3	*Ardeola bacchus*	faeces	Leizhou	*blaCARB-33*	none	none	none	*tlh*	24	2	0.016	0.19	0.094	0.75	0.094	2	8	32	1	1	1	0.38	0.25	0.38	0.19	0.19	JAQBME000000000
L14-1	2019.3	*Ardeola bacchus*	faeces	Leizhou	*blaCARB-26*	none	IS1595,IS200_IS605,IS4,IS481,ISL3,ISNCY	none	*tlh*	16	6	0.016	0.19	0.047	0.5	0.125	2	4	32	1	1	1	0.38	0.25	0.38	0.19	0.125	JAQBMF000000000
L14-2	2019.3	*Ardeola bacchus*	faeces	Leizhou	*blaCARB-26*	none	IS1595,IS4,IS481,ISL3,ISNCY	none	*tlh*	16	6	0.016	0.19	0.094	0.5	0.094	2	2	12	1	2	1	0.19	0.25	0.38	0.19	0.125	JAQBMG000000000
L15-1	2019.3	*Ardeola bacchus*	faeces	Leizhou	*blaCARB-33*	none	none	none	*tlh*	8	2	0.016	0.19	0.25	0.25	0.125	2	6	24	1	2	0.75	0.38	0.125	0.38	0.38	0.25	JAQBMH000000000
L20-1	2019.3	*Ardeola bacchus*	faeces	Leizhou	*blaCARB-33*	none	none	none	*tlh*	16	6	0.016	0.19	0.094	0.25	0.094	2	2	12	0.5	2	1	0.38	0.25	0.38	0.19	0.19	JAQBMI000000000
L21-1	2019.3	*Ardeola bacchus*	faeces	Leizhou	*blaCARB-20*	none	ISL3	none	*tlh*	16	3	0.016	0.19	2	4	4	2	6	32	1	2	1	0.38	0.38	0.19	0.19	0.19	JAQBMJ000000000
L23-1	2019.3	*Charadriiformes*	faeces	Leizhou	*blaCARB-26*	none	IS1595,IS4,IS481,ISL3,ISNCY	none	*tlh*	16	6	0.016	0.19	1.5	4	4	2	6	32	1	1	1	0.38	0.25	0.38	0.19	0.19	JAQBMK000000000
L23-2	2019.3	*Charadriiformes*	faeces	Leizhou	*blaCARB-26*	none	IS1595,IS4,IS481,ISL3,ISNCY	none	*tlh*	8	3	0.016	0.38	0.094	0.5	0.38	2	6	24	1	1	1	0.38	0.25	0.19	0.19	0.25	JAQBML000000000
L26-1	2019.3	*Charadriiformes*	faeces	Leizhou	*blaCARB-26, sul2, tet(59)*	none	IS91,IS1595,IS200_IS605,IS30,IS4,IS481,IS5,IS66	none	*tlh*	16	6	0.016	0.19	0.094	0.5	0.094	2	6	32	1	3	1	0.38	0.25	0.38	0.19	0.19	JAQBMM000000000
L28-1	2019.3	*Charadriiformes*	faeces	Leizhou	*blaCARB-25, blaCARB-29, blaCARB-33, blaCARB-41*	none	IS1595,IS3,IS4,IS481,ISL3,ISNCY	none	*tlh*	16	6	0.016	0.19	0.094	0.5	0.094	2	2	6	0.5	1	1	0.38	0.25	0.38	0.19	0.19	JAQBMN000000000
L29-1	2019.3	*Charadriiformes*	faeces	Leizhou	*blaCARB-26*	none	IS1959,IS4,IS481，ISL3，ISNCY	none	*tlh*	16	6	0.016	0.19	0.094	0.75	0.094	2	4	24	1	2	1	0.38	0.25	0.38	0.19	0.125	JAQBMO000000000

† OCTWP=Overseas Chinese Town Wetland Park; FRFR=Futian Red Forest Reserve

& AK=Amikacin；GM=Gentamicin; MEM=Meropenem; IP=Imipenem; CTX=Cefotaxime; FEP=Cefepime; CAZ=Ceftazidime; ATM=Aztreonam; AC=Amoxicillin; AMP=Ampicillin; AMC=Amoxicillin/clavulanic acid; SAM=Ampicillin/sulbactam; C=Chloramphenicol; CIP=Ciprofloxacin; LVX=Levofloxacin; MXF=Moxifloxacin; TE=Tetracycline; SXT=Trimethoprim/sulfamethoxazole

The yellow area: the MIC value of the strains showed intermediate resistance to antibiotics

The red area: the MIC value of the strains showed resistance to antibiotics

The white area: the MIC value of the strains showed sensitive to antibiotics

The blue area: CLSI standards were not applicable, it was impossible to determine whether they were resistant or sensitive

Supplementary Table 2 MLST details of 124 *V. parahaemolyticus* strains in this study

Isolate name	dnaE	gyrB	recA	dtds	pntA	pyrC	tnaA	ST
FT2-2<br>FT3-1	3	57	3	55	304	486	61	ST-2710
1-2<br>6-1	3	106	173	186	78	11	264	ST-1869
1-1	3	131	249	126	28	226	159	ST-1339
8-1	5	380	59	533	4	45	47	ST-2712
21-2	5	524	450	401	56	468	24	ST-2711
SH14-2	6	200	3	17	23	65	26	ST-2692
5-2	7	23	81	224	43	45	314	ST-2714
WF2-1<br>WF2-2	7	302	4	26	6	18	26	ST-634
28-1	19	217	89	112	26	62	319	ST-2715
26-2<br>33-1<br>33-2	19	217	451	112	26	62	319	ST-2709
FT2-1	19	392	443	14	156	11	71	ST-2717
F14-1<br>F14-2	31	106	135	74	37	212	54	ST-564
28-2	20	153	452	263	50	157	23	ST-2719
SH11-1	22	254	75	205	117	488	26	ST-2720
36-1<br>37-2<br>40-2<br>41-2	26	354	82	251	18	69	13	ST-2693
38-1	28	4	82	88	63	187	1	ST-799
36-2<br>41-1	31	13	129	180	128	62	23	ST-2695
14-2	31	106	135	74	37	212	54	ST-564
9-1<br>9-2	31	377	268	224	26	73	23	ST-972
10-1<br>10-2	31	593	91	529	31	45	62	ST-2687
18-2	35	154	31	78	82	485	258	ST-2721
Z13-1<br>Z13-2<br>Z14-2<br>Z19-1	35	43	38	21	31	35	37	ST-79
11-2<br>13-1<br>14-1	35	594	440	491	195	180	62	ST-2688
3-2	42	348	178	129	50	494	81	ST-2722
42-2	42	298	31	531	234	186	87	ST-2723
WS1-1	42	298	31	19	224	171	87	ST2696
FT8-1	42	319	123	151	161	61	24	ST-705
FT8-2	42	599	444	378	276	487	316	ST-2724
2-1								

Isolate name	dnaE	gyrB	recA	dtds	pntA	pyrC	tnaA	ST
27-1	44	73	181	530	18	45	51	ST-2725
42-1								
7-2	44	130	87	110	56	37	63	ST-2097
5-1	47	58	53	19	50	37	26	ST-162
L2-1								
L15-1	51	4	77	84	60	8	33	ST-658
L20-1								
8-2	51	4	77	76	60	173	33	ST-2423
24-1	51	29	77	13	60	8	33	ST-356
11-1	51	555	61	76	23	457	76	ST-2445
Z11-1	60	49	89	539	18	216	318	ST-2726
7-1	60	67	4	53	43	63	23	ST-104
Z14-1	60	186	287	351	78	36	226	ST-2697
25-2	71	257	183	9	132	26	26	ST-2698
Z11-2	92	261	25	540	50	73	26	ST-2727
21-1	103	3	89	3	72	82	2	ST-200
34-1	111	602	453	541	306	71	248	ST-2728
SH15-1	112	104	23	447	18	490	26	ST-2729
L21-1	116	147	89	76	45	184	26	ST-2699
29-2	123	100	127	29	66	11	31	ST-2026
40-1	123	232	61	73	26	45	57	ST-2700
SH8-2	132	600	31	350	28	491	26	ST-2730
SH2-1								
SH2-2	133	136	3	537	43	63	12	ST-2731
SH4-3								
39-1	137	124	411	76	128	194	195	ST-2701
35-1	139	8	78	543	60	69	57	ST-2732
35-2								
SH5-1	144	92	69	114	54	71	192	ST-804
31-2	148	8	181	74	142	8	320	ST-2733
17-1	153	191	70	19	23	8	1	ST-355
38-2	154	603	74	544	78	495	132	ST-2734
23-1	158	23	153	74	66	493	33	ST-2735
34-2	167	68	454	542	307	46	13	ST-2736
SH10-3	175	530	168	169	130	17	73	ST-2126
SH10-4	175	598	25	150	26	78	258	ST-2752
F7-2								
L14-1								
L14-2	175	598	25	150	26	78	33	ST-2738
L23-1								
L23-2								
L29-1								
F7-1	175	597	89	29	18	96	140	ST-2737
L28-1	175	598	445	13	26	78	33	ST-2739
SH7-2	179	232	58	27	125	5	26	ST-2702
12-1	185	217	441	151	2	37	9	ST-2689
25-1	190	15	31	55	18	58	23	ST-1353

Isolate name	dnaE	gyrB	recA	dtds	pntA	pyrC	tnaA	ST
SH8-1<br>SH15-2<br>3-1	191	187	446	185	4	77	57	ST-2740
4-1<br>4-2	193	261	109	106	50	10	23	ST-2703
13-2	221	298	31	55	26	23	37	ST-2690
20-2	236	25	449	74	43	231	12	ST-2741
15-2	250	2	59	309	26	11	26	ST-2691
SH13-1	265	371	273	217	23	238	146	ST-2706
23-2	291	177	19	125	18	175	26	ST-2704
L26-1	305	139	24	5	195	27	94	ST-1275
29-1	356	25	145	27	244	343	17	ST-2705
SH14-1<br>31-1<br>32-1<br>32-2<br>SH7-1	356	230	72	535	63	489	105	ST-2742
16-1<br>18-1<br>26-1	366	498	102	90	26	319	207	ST-2707
15-1	372	357	361	85	6	37	24	ST-1865
24-2	382	136	61	180	31	212	54	ST-2708
37-1	442	4	70	191	165	17	29	ST-2751
16-2	436	82	67	110	18	11	54	ST-2744
SH16-1<br>SH16-2	439	89	25	536	47	3	38	ST-2748
20-1	440	285	448	19	26	133	84	ST-2749
FT3-2	438	408	3	110	305	393	315	ST-2746
Z8-1	438	408	178	110	26	244	315	ST-2747
6-2	437	595	53	532	50	37	12	ST-2745
27-2	441	601	194	89	26	46	143	ST-2750

Supplementary Table 3 The information of each 464 non-bird-carried representative *V. parahaemolyticus* strains

Type	Strain ID	Year	Location	Sample type	Source	Population	Accession Number
Gly-4TR	RIMD 2210633	1996	Japan	Clinical	Stool	VppAsia	GCA_000196095.1
Gly-4TR	Ba94C2	2015	NA	Environmental	Shellfish	VppAsia	SRR3987381
Gly-4TR	TUMSAT_H10_S6	NA	Thailand	Environmental	Shellfish	VppAsia	GCA_000591555.1
Gly-4TR	TUMSAT_D06_S3	NA	Thailand	Environmental	Shellfish	VppAsia	GCA_000591495.1
Gly-4TR	EKP-008	NA	NA	Environmental	NA	VppAsia	GCA_000510585.2
Gly-4TR	CDC_K4557	2006	USA: LA	Clinical	Stool	VppAsia	GCA_000430425.1
Gly-4TR	CFSAN007444	2012	USA: MD	Clinical	Wound	VppAsia	GCA_000707065.2
Gly-4TR	CFSAN007457	2010	USA: MD	Environmental	Shellfish	VppX	GCA_000707085.2
Gly-4TR	CFSAN007459	2010	USA: MD	Environmental	Shellfish	VppX	GCA_000707185.2
Gly-4TR	13-028A3	2013	Vietnam	Environmental	Shellfish	VppAsia	GCA_000737635.1
Gly-4TR	NCKU_TV_5HP	1999	Thailand	Environmental	Shellfish	VppAsia	GCA_000736315.1
Gly-4TR	NCKU_CV_CHN	2010	China	Environmental	Shellfish	VppX	GCA_000736325.1
Gly-4TR	UCM-V493	NA	spain	Environmental	NA	VppAsia	GCA_000568495.1
Gly-4TR	FORC_004	2014	SouthKorea: Busan	Environmental	Water	VppX	GCA_001433415.1
Gly-4TR	FORC_006	2014	SouthKorea: Gyeongnam	Environmental	Others	VppAsia	GCA_001304775.1
Gly-4TR	FORC_008	2014	SouthKorea: Gyeongnam	Environmental	Fish	VppAsia	GCA_001244315.1
Gly-4TR	T9109	2007	Canada: British Columbia	Clinical	NA	VppAsia	GCA_000786845.1
Gly-4TR	CHN25	2011	China: Shanghai	Environmental	NA	VppAsia	GCA_001700835.1
Gly-4TR	07-2965	2007	Canada: Alberta	Clinical	NA	VppAsia	GCA_000960565.1
Gly-4TR	09-4681	2009	Canada: New Brunswick	Clinical	NA	VppX	GCA_000972025.1
Gly-4TR	FORC_023	2014	SouthKorea: Pusan	Clinical	Others	VppAsia	GCA_001758605.1
Gly-4TR	ISF-25-6	2010	Canada	Environmental	Shellfish	VppX	GCA_001267595.1
Gly-4TR	RM-13-3	2015	Canada	Environmental	Shellfish	VppAsia	GCA_001267965.1
Gly-4TR	RM-17-6	2015	Canada	Environmental	Shellfish	VppX	GCA_001267655.1
Gly-4TR	S195-7	2007	Canada	Environmental	Others	VppX	GCA_001268005.1
Gly-4TR	S349-10	2010	Canada	Environmental	Others	VppAsia	GCA_001268015.1
Gly-4TR	S383-6	2011	Canada	Environmental	Others	VppX	GCA_001267625.1
Gly-4TR	ISF-29-3	2011	Canada	Environmental	Shellfish	VppAsia	GCA_001273575.1
Gly-4TR	ISF-77-01	2011	Canada	Environmental	Shellfish	VppAsia	GCA_001270285.1
Gly-4TR	ISF-94-1	2011	Canada	Environmental	Shellfish	VppAsia	GCA_001280645.1
Gly-4TR	S487-4	2013	Canada	Environmental	Shellfish	VppAsia	GCA_001270215.1
Gly-4TR	CFSAN018755	2002	Spain	Environmental	NA	VppAsia	GCA_001695935.1
Gly-4TR	CFSAN018756	2003	Spain	Environmental	NA	VppAsia	GCA_001696005.1
Gly-4TR	CFSAN018758	1999	Peru	Clinical	Stool	VppAsia	GCA_001696055.1
Gly-4TR	CFSAN018765	2004	USA	Environmental	NA	VppX	GCA_001696165.1
Gly-4TR	NSV_5736	NA	USA	Clinical	NA	VppX	GCA_001471485.2
Gly-4TR	FORC_022	NA	SouthKorea	Environmental	Shellfish	VppAsia	GCA_001879585.1
Gly-4TR	A4EZ703	2004	Canada: British Columbia	Clinical	Stool	VppX	GCA_001584415.1
Gly-4TR	05-4792	2005	Canada: Alberta	Clinical	Stool	VppX	GCA_001609435.1
Gly-4TR	C150	2008	Canada: British Columbia	Clinical	Stool	VppAsia	GCA_001601695.1
Gly-4TR	04-2192	2004	Canada: Saskatchewan	Clinical	Stool	VppAsia	GCA_001609555.1
Gly-4TR	04-2550	2004	Canada: Ontario	Clinical	Stool	VppX	GCA_001610595.1
Gly-4TR	A3EZ936	2003	Canada: British Columbia	Clinical	Stool	VppAsia	GCA_001609175.1
Gly-4TR	1682	2004	Norway	Clinical	Wound	VppAsia	GCA_001951115.1
Gly-4TR	MVP1	2016	Malaysia: Negeri Sembilan	Environmental	NA	VppAsia	GCA_001996365.1
Gly-4TR	GIMxtf283-2012.12	2012	China: Shenzhen	Environmental	Shellfish	VppAsia	GCA_001913695.1
Gly-4TR	GIMxtfL88-2011.05	2011	China: Shenzhen	Clinical	Stool	VppAsia	GCA_001921805.1
Gly-4TR	PY233	2006	Spain	Environmental	NA	VppAsia	SRR5163844
Gly-4TR	OJL90_2nd	2007	Spain	Environmental	NA	VppAsia	SRR6410788
Gly-4TR	CFSAN045070_113477	2015	Spain: Galicia	Clinical	NA	VppAsia	SRR5163834
Gly-4TR	14-1072-D-VP	NA	NA	NA	NA	VppAsia	SRR5639920
Gly-4TR	C21-1262-07	2007	Peru	Clinical	NA	VppAsia	SRR6410782
Gly-4TR	A3_6	2014	China: Henan	Environmental	Fish	VppAsia	NNQM00000000
Gly-4TR	B1_7	2014	China: Hubei	Environmental	Fish	VppAsia	NNPQ00000000
Gly-4TR	B2_10	2014	China: Hubei	Environmental	Fish	VppAsia	NNPE00000000
Gly-4TR	B5_6	2014	China: Hubei	Environmental	Fish	VppAsia	NNNW00000000
Gly-4TR	C10_4	2014	China: Zhejiang	Environmental	Water	VppAsia	NNMA00000000
Gly-4TR	C3_7	2014	China: Shandong	Environmental	Water	VppAsia	NNLN00000000
Gly-4TR	C3_9	2014	China: Shandong	Environmental	Water	VppAsia	NNLM00000000
Gly-4TR	C6_8	2014	China: Shandong	Environmental	Fish	VppAsia	NNKL00000000
Gly-4TR	D1_1	2014	China: Sichuan	Environmental	Sediment	VppAsia	NNJT00000000
Gly-4TR	D1_7	2014	China: Sichuan	Environmental	Fish	VppAsia	NNJQ00000000
Gly-4TR	E10_9	2014	China: Zhejiang	Environmental	Shellfish	VppAsia	NNHO00000000
Gly-4TR	E4_2	2014	China: Zhejiang	Environmental	Fish	VppX	NNHG00000000
Gly-4TR	F10_3	2014	China: Guangxi	Environmental	Fish	VppAsia	NNGJ00000000
Gly-4TR	F10_9	2014	China: Guangxi	Environmental	Fish	VppAsia	NNGD00000000
Gly-4TR	F6_2	2014	China: Guangxi	Environmental	Water	VppAsia	NNEO00000000

Type	Strain ID	Year	Location	Sample type	Source	Population	Accession Number
Gly-4TR	F6_3	2014	China: Guangxi	Environmental	Water	VppAsia	NNEN00000000
Gly-4TR	G1_1	2014	China: Guangxi	Environmental	Fish	VppAsia	NNDE00000000
Gly-4TR	ATCC-17802	1951	Japan	Environmental	Fish	VppAsia	GCA_001558495.2
Other	CFSAN018752	1998	Spain	Clinical	Stool	VppAsia	GCA_001695955.1
Other	TUMSAT_H01_S4	NA	Thailand	Environmental	Shellfish	VppUS1	GCA_000591515.1
Other	VP2007-007	NA	NA	Environmental	NA	VppUS1	GCA_000558925.2
Other	VPTS-2009	NA	NA	Environmental	NA	VppAsia	GCA_000593325.2
Other	AQ3810	NA	NA	Environmental	NA	VppAsia	GCA_000582905.2
Other	VPTS-2010	NA	NA	Environmental	NA	VppAsia	GCA_000593345.2
Other	VPTS-2010_2	NA	NA	Environmental	NA	VppUS2	GCA_000593365.2
Other	VPCR-2009	NA	NA	Environmental	NA	VppAsia	GCA_000593305.2
Other	FDA_R31	2007	USA: LA	Environmental	Shellfish	VppUS2	GCA_000430405.1
Other	S001	NA	Thailand	Clinical	NA	VppAsia	GCA_000492095.1
Other	S002	1999	China: Taiwan	Clinical	NA	VppAsia	GCA_000492075.1
Other	S015	1992	China: Taiwan	Clinical	NA	VppAsia	GCA_000491875.1
Other	S017	1992	China: Taiwan	Clinical	NA	VppAsia	GCA_000491835.1
Other	S022	1984	Japan	Clinical	NA	VppAsia	GCA_000491735.1
Other	S023	1994	China: Taiwan	Clinical	NA	VppAsia	GCA_000491715.1
Other	S024	1998	China: Taiwan	Clinical	NA	VppAsia	GCA_000491695.1
Other	S028	NA	Thailand	Clinical	NA	VppAsia	GCA_000491615.1
Other	S029	1981	Spain	Environmental	NA	VppAsia	GCA_000491595.1
Other	S039	NA	China: Taiwan	Clinical	NA	VppAsia	GCA_000491395.1
Other	S040	1990	Thailand	Clinical	NA	VppAsia	GCA_000491375.1
Other	S042	1984	Japan	Clinical	NA	VppAsia	GCA_000491335.1
Other	S043	NA	Thailand	Clinical	NA	VppAsia	GCA_000491315.1
Other	S046	1982	Spain	Environmental	NA	VppX	GCA_000491255.1
Other	S047	1981	Spain	Environmental	NA	VppAsia	GCA_000491235.1
Other	S048	1997	USA	Environmental	NA	VppX	GCA_000491215.1
Other	S052	1975	Spain	Environmental	NA	VppUS1	GCA_000491175.1
Other	S053	1990	Thailand	Clinical	NA	VppAsia	GCA_000491155.1
Other	S058	1970	Japan	Clinical	NA	VppAsia	GCA_000491055.1
Other	S060	1992	China: Taiwan	Clinical	NA	VppAsia	GCA_000491035.1
Other	S093	1998	Japan	Clinical	NA	VppAsia	GCA_000490485.1
Other	S096	1999	Korea	Clinical	NA	VppAsia	GCA_000493225.1
Other	S097	1992	China: Taiwan	Clinical	NA	VppX	GCA_000490415.1
Other	S100	1990	USA	Environmental	NA	VppUS1	GCA_000490375.1
Other	S105	1984	Japan	Clinical	NA	VppAsia	GCA_000490315.1
Other	S108	1990	Thailand	Clinical	NA	VppAsia	GCA_000490275.1
Other	S115	1991	Thailand	Clinical	NA	VppAsia	GCA_000490135.1
Other	S118	1984	Japan	Clinical	NA	VppAsia	GCA_000490075.1
Other	S119	1999	China: Taiwan	Clinical	NA	VppAsia	GCA_000490055.1
Other	S121	NA	Thailand	Clinical	NA	VppAsia	GCA_000490015.1
Other	S122	1999	India	Clinical	NA	VppAsia	GCA_000489995.1
Other	S128	1999	India	Clinical	NA	VppAsia	GCA_000489895.1
Other	S134	2005	China: Liaoning	Clinical	NA	VppAsia	GCA_000489775.1
Other	S139	2006	China: inter Mongolia	Environmental	NA	VppAsia	GCA_000489675.1
Other	S140	2006	China: Shandong	Environmental	NA	VppAsia	GCA_000489655.1
Other	S141	2006	China: Shandong	Environmental	NA	VppAsia	GCA_000489635.1
Other	S142	2006	China: Shandong	Environmental	NA	VppAsia	GCA_000489615.1
Other	S145	2006	China: inter Mongolia	Environmental	NA	VppAsia	GCA_000489555.1
Other	S146	2006	China: inter Mongolia	Environmental	NA	VppAsia	GCA_000489535.1
Other	S147	2006	China: inter Mongolia	Environmental	NA	VppAsia	GCA_000489515.1
Other	S148	2006	China: inter Mongolia	Environmental	NA	VppAsia	GCA_000489495.1
Other	S152	2006	China: Shandong	Environmental	NA	VppAsia	GCA_000489435.1
Other	S159	2006	China: Shandong	Environmental	NA	VppAsia	GCA_000489295.1
Other	S161	2006	China: Shandong	Environmental	NA	VppAsia	GCA_000489255.1
Other	S162	2007	Thailand	Environmental	NA	VppAsia	GCA_000489235.1
Other	S164	2007	USA	Environmental	NA	VppAsia	GCA_000489195.1
Other	S165	2007	China: Shandong	Environmental	NA	VppAsia	GCA_000489175.1
Other	S166	2007	China: Shandong	Environmental	NA	VppAsia	GCA_000489155.1
Other	S168	2007	China: Shandong	Environmental	NA	VppAsia	GCA_000489115.1
Other	S171	2007	China: Shandong	Environmental	NA	VppAsia	GCA_000489075.1
Other	S172	2007	China: Shandong	Environmental	NA	VppAsia	GCA_000489055.1
Other	S173	2007	China: Shandong	Environmental	NA	VppAsia	GCA_000489035.1
Other	S174	2007	China: Shandong	Environmental	NA	VppAsia	GCA_000489015.1
Other	GCSL_R7	2007	USA: TX	Environmental	Shellfish	VppX	GCA_001726195.1
Other	GCSL_R13	2007	USA: LA	Environmental	Shellfish	VppAsia	GCA_001726255.1
Other	GCSL_R17	2007	USA: FL	Environmental	Shellfish	VppAsia	GCA_001726275.1

Type	Strain ID	Year	Location	Sample type	Source	Population	Accession Number
Other	GCSL_R26	2007	USA: NJ	Environmental	Shellfish	VppUS1	GCA_001726335.1
Other	GCSL_R29	2007	USA: FL	Environmental	Shellfish	VppUS1	GCA_001726325.1
Other	GCSL_R32	2007	USA: LA	Environmental	Shellfish	VppUS2	GCA_001726445.1
Other	GCSL_R45	2007	USA: WA	Environmental	Shellfish	VppUS1	GCA_001726495.1
Other	GCSL_R52	2007	USA: WA	Environmental	Shellfish	VppAsia	GCA_001726565.1
Other	GCSL_R60	2007	USA: ME	Environmental	Shellfish	VppAsia	GCA_001726695.1
Other	GCSL_R62	2007	USA: ME	Environmental	Shellfish	VppUS2	GCA_001726725.1
Other	GCSL_R63	2007	USA: ME	Environmental	Shellfish	VppAsia	GCA_001726735.1
Other	GCSL_R65	2007	USA: ME	Environmental	Shellfish	VppUS2	GCA_001726755.1
Other	GCSL_R74	2007	USA: VA	Environmental	Shellfish	VppAsia	GCA_001726775.1
Other	GCSL_R77	2007	USA: VA	Environmental	Shellfish	VppAsia	GCA_001726845.1
Other	GCSL_R86	2007	USA: FL	Environmental	Shellfish	VppUS1	GCA_001726885.1
Other	GCSL_R88	2007	USA: FL	Environmental	Shellfish	VppAsia	GCA_001726895.1
Other	GCSL_R111	2007	Canada: PEI	Environmental	Shellfish	VppUS1	GCA_001727065.1
Other	GCSL_R125	2007	USA: FL	Environmental	Shellfish	VppUS2	GCA_001727045.1
Other	GCSL_R126	2007	USA: FL	Environmental	Shellfish	VppAsia	GCA_001727105.1
Other	GCSL_R129	2007	USA: FL	Environmental	Shellfish	VppUS2	GCA_001727115.1
Other	GCSL_R130	2007	USA: FL	Environmental	Shellfish	VppAsia	GCA_001975475.1
Other	GCSL_R131	2007	USA: FL	Environmental	Shellfish	VppX	GCA_001727125.1
Other	GCSL_R135	2007	USA: SC	Environmental	Shellfish	VppAsia	GCA_001727155.1
Other	GCSL_R138	2007	USA: SC	Environmental	Shellfish	VppUS2	GCA_001727205.1
Other	GCSL_R143	2007	USA: FL	Environmental	Shellfish	VppUS1	GCA_001727265.1
Other	GCSL_R145	2007	USA: FL	Environmental	Shellfish	VppUS1	GCA_001727295.1
Other	CDC_K4556R	2006	USA: LA	Clinical	Wound	VppAsia	GCA_001727365.1
Other	CDC_K4558G	2006	USA: LA	Clinical	Wound	VppUS1	GCA_001727735.1
Other	CDC_K4764D	2006	USA: VA	Clinical	Stool	VppAsia	GCA_001727975.1
Other	CDC_K4842	2006	USA: MD	Clinical	Stool	VppAsia	GCA_001727995.1
Other	CDC_K4857G	2007	USA: HI	Clinical	Stool	VppAsia	GCA_001728045.1
Other	CDC_K4858	2006	USA: HI	Clinical	Stool	VppAsia	GCA_001728085.1
Other	CDC_K4981	2007	USA: OK	Clinical	NA	VppUS2	GCA_001728125.1
Other	CDC_K5059W	2007	USA: TX	Clinical	NA	VppAsia	GCA_001728235.1
Other	CDC_K5125	2007	USA: MS	Clinical	NA	VppAsia	GCA_001728295.1
Other	CDC_K5126	2007	USA: MS	Clinical	Stool	VppUS1	GCA_001728325.1
Other	CDC_K5323G	NA	USA: VA	Clinical	NA	VppAsia	GCA_001727475.1
Other	CDC_K5324W	2007	USA: VA	Clinical	Stool	VppUS2	GCA_001727405.1
Other	CDC_K5328	NA	USA: IN	Clinical	Stool	VppX	GCA_001727665.1
Other	CDC_K5635	2007	USA: MD	Clinical	Wound	VppX	GCA_001728725.1
Other	970107	1997	USA	Environmental	Water	VppAsia	GCA_000510605.2
Other	PCV08-7	2008	Malaysia: Selangor	Environmental	Others	VppAsia	GCA_000347495.1
Other	VIP4-0219	2006	China: Hong Kong	Environmental	Fish	VppAsia	GCA_000500525.1
Other	VIP4-0444	2008	China: Hong Kong	Environmental	Fish	VppAsia	GCA_000500485.1
Other	VIP4-0430	2008	China: Hong Kong	Environmental	Shellfish	VppAsia	GCA_000500445.1
Other	VIP4-0447	2008	China: Hong Kong	Environmental	Shellfish	VppAsia	GCA_000500545.1
Other	v110	2010	China: Hong Kong	Environmental	Shellfish	VppAsia	GCA_000388025.1
Other	M0605	2013	Mexico: Sinaloa	Environmental	Shellfish	VppX	GCA_000523375.1
Other	VP49	2008	India: Mangalore	Environmental	Others	VppAsia	GCA_000662375.1
Other	K1275	2004	USA: TX	Clinical	NA	VppAsia	GCA_000958585.1
Other	SG176	2006	USA: GA	Environmental	NA	VppAsia	GCA_000958565.1
Other	J-C2-34	1998	USA: NC	Environmental	NA	VppUS2	GCA_000958655.1
Other	22702	1998	USA: GA	Environmental	NA	VppUS2	GCA_000958645.1
Other	CFSAN007437	2012	USA: MD	Clinical	Stool	VppX	GCA_000707165.2
Other	CFSAN007438	2012	USA: MD	Clinical	Wound	VppAsia	GCA_000707145.2
Other	CFSAN007439	2012	USA: DE	Clinical	Stool	VppUS1	GCA_000707025.2
Other	CFSAN007446	2012	USA: MD	Clinical	Wound	VppAsia	GCA_000707685.2
Other	CFSAN007447	2012	USA: MD	Clinical	Others	VppAsia	GCA_000707705.2
Other	CFSAN007455	2013	USA: MD	Clinical	Stool	VppX	GCA_000706985.2
Other	CFSAN007456	2010	USA: MD	Environmental	Shellfish	VppAsia	GCA_000707285.2
Other	CFSAN012492	2010	USA: MD	Environmental	Shellfish	VppAsia	GCA_000707665.2
Other	13-028A2	2013	Vietnam	Environmental	Shellfish	VppAsia	GCA_000732245.1
Other	NCKU_TN_S02	2008	Thailand	Environmental	Sediment	VppAsia	GCA_000736345.1
Other	FIM-S1392-	2014	Mexico: Hermosillo	Environmental	Sediment	VppAsia	GCA_000732995.1
Other	MAVP-M	2011	NA	Clinical	NA	VppAsia	GCA_001023155.1
Other	04.2548	2004	Canada: Ontario	Clinical	NA	VppAsia	GCA_000786855.1
Other	09.5357	2009	Canada: New Brunswick	Clinical	NA	VppAsia	GCA_000786865.1
Other	09-3217	2009	Canada: British Columbia	Clinical	NA	VppUS2	GCA_000960655.1
Other	GCSL_R5	2007	USA: TX	Environmental	Shellfish	VppAsia	GCA_001726165.1
Other	GCSL_R10	2007	USA: FL	Environmental	Shellfish	VppUS2	GCA_001726175.1
Other	GCSL_R42	2007	USA: WA	Environmental	Shellfish	VppUS1	GCA_001726485.1

Type	Strain ID	Year	Location	Sample type	Source	Population	Accession Number
Other	GCSL_R59	2007	USA: ME	Environmental	Shellfish	VppUS1	GCA_001726685.1
Other	GCSL_R98	2007	Canada: PEI	Environmental	Shellfish	VppAsia	GCA_001726965.1
Other	CDC_K4764L	2006	USA: VA	Clinical	Stool	VppAsia	GCA_001727965.1
Other	CDC_K5330	2007	USA: TX	Clinical	NA	VppAsia	GCA_001728375.1
Other	CDC_K5620	2007	USA: NY	Clinical	Stool	VppX	GCA_001728665.1
Other	CDC_K5701	2007	USA: OR	Clinical	Stool	VppAsia	GCA_001728755.1
Other	FORC_014	2015	SouthKorea: Pusan	Environmental	NA	VppAsia	GCA_001636035.1
Other	VH3	2007	Greece	Environmental	Water	VppAsia	GCA_001013435.1
Other	K23	2013	India	Environmental	Others	VppAsia	GCA_001497485.1
Other	RM-14-5	2014	Canada	Environmental	Shellfish	VppUS1	GCA_001273555.1
Other	ISF-54-12	2011	Canada	Environmental	Shellfish	VppUS2	GCA_001280635.1
Other	S357-21	2010	Canada	Environmental	Shellfish	VppUS2	GCA_001273635.1
Other	S440-7	2012	Canada	Environmental	Shellfish	VppUS1	GCA_001270235.1
Other	CFSAN018766	2004	USA	Environmental	NA	VppAsia	GCA_001696215.1
Other	CFSAN018767	2004	USA	Environmental	NA	VppUS2	GCA_001696225.1
Other	CFSAN025056	2009	Peru	Clinical	NA	VppAsia	GCA_001634185.1
Other	A5Z1022	2005	Canada: British Columbia	Clinical	Stool	VppAsia	GCA_001608755.1
Other	W501	2006	Canada: British Columbia	Clinical	Stool	VppAsia	GCA_001608855.1
Other	A0EZ383	2000	Canada: British Columbia	Clinical	Stool	VppAsia	GCA_001609055.1
Other	06-043	2006	Mozambique	Environmental	NA	VppAsia	GCA_001856035.1
Other	KVp10	2007	Sweden	Environmental	NA	VppUS2	GCA_001707995.1
Other	R10B2_71	1997	USA: WA	Environmental	Shellfish	VppUS2	GCA_001704915.1
Other	GIMxtf381-2013.06	2013	China: Haikou	Environmental	Fish	VppAsia	GCA_001913785.1
Other	OAG95	2007	Spain	Environmental	NA	VppX	SRR5163851
Other	G32	2012	Spain: Galicia	Clinical	NA	VppAsia	SRR5163840
Other	CFSAN045069_119	2015	Spain: Galicia	Clinical	NA	VppAsia	SRR5163836
Other	14-1499-VP	2014	UK	Environmental	Shellfish	VppUS2	SRR5639914
Other	14-1498-F-VP	2014	UK	Environmental	Shellfish	VppUS2	SRR5639919
Other	A1_2	2014	China: Henan	Environmental	Fish	VppAsia	NNRG00000000
Other	A1_6	2014	China: Henan	Environmental	Fish	VppAsia	NNRD00000000
Other	A1_7	2014	China: Henan	Environmental	Fish	VppX	NNRC00000000
Other	A1_8	2014	China: Henan	Environmental	Fish	VppX	NNRB00000000
Other	A1_9	2014	China: Henan	Environmental	Water	VppAsia	NNRA00000000
Other	A2_1	2014	China: Henan	Environmental	Fish	VppAsia	NNQZ00000000
Other	A2_2	2014	China: Henan	Environmental	Fish	VppX	NNQY00000000
Other	A2_4	2014	China: Henan	Environmental	Fish	VppAsia	NNQW00000000
Other	A2_5	2014	China: Henan	Environmental	Fish	VppAsia	NNQV00000000
Other	A2_6	2014	China: Henan	Environmental	Fish	VppAsia	NNQU00000000
Other	A2_7	2014	China: Henan	Environmental	Water	VppAsia	NNQT00000000
Other	A2_8	2014	China: Henan	Environmental	Fish	VppAsia	NNQS00000000
Other	A3_2	2014	China: Henan	Environmental	Fish	VppAsia	NNQQ00000000
Other	A3_4	2014	China: Henan	Environmental	Fish	VppAsia	NNQO00000000
Other	A3_5	2014	China: Henan	Environmental	Water	VppX	NNQN00000000
Other	A3_7	2014	China: Henan	Environmental	Fish	VppAsia	NNQL00000000
Other	A3_9	2014	China: Henan	Environmental	Fish	VppAsia	NNQJ00000000
Other	A4_1	2014	China: Henan	Environmental	Fish	VppAsia	NNQI00000000
Other	A4_4	2014	China: Henan	Environmental	Fish	VppAsia	NNQF00000000
Other	A4_5	2014	China: Henan	Environmental	Fish	VppAsia	NNQE00000000
Other	A4_6	2014	China: Henan	Environmental	Fish	VppAsia	NNQD00000000
Other	A4_9	2014	China: Henan	Environmental	Water	VppAsia	NNQC00000000
Other	A5_3	2014	China: Henan	Environmental	Water	VppAsia	NNQA00000000
Other	A5_4	2014	China: Henan	Environmental	Fish	VppAsia	NNPZ00000000
Other	A5_7	2014	China: Henan	Environmental	Fish	VppAsia	NNPY00000000
Other	A5_8	2014	China: Henan	Environmental	Fish	VppAsia	NNPX00000000
Other	B1_1	2014	China: Hubei	Environmental	Fish	VppAsia	NNPW00000000
Other	B1_10	2014	China: Hubei	Environmental	Fish	VppAsia	NNPV00000000
Other	B1_3	2014	China: Hubei	Environmental	Fish	VppAsia	NNPU00000000
Other	B1_5	2014	China: Hubei	Environmental	Fish	VppAsia	NNPS00000000
Other	B1_8	2014	China: Hubei	Environmental	Fish	VppAsia	NNPP00000000
Other	B10_1	2014	China: Hubei	Environmental	Fish	VppAsia	NNPN00000000
Other	B10_2	2014	China: Hubei	Environmental	Fish	VppX	NNPM00000000
Other	B10_3	2014	China: Hubei	Environmental	Fish	VppAsia	NNPL00000000
Other	B10_7	2014	China: Hubei	Environmental	Fish	VppAsia	NNPI00000000
Other	B2_4	2014	China: Hubei	Environmental	Fish	VppAsia	NNPB00000000
Other	B2_8	2014	China: Hubei	Environmental	Fish	VppAsia	NNOX00000000
Other	B3_1	2014	China: Hubei	Environmental	Fish	VppAsia	NNOV00000000
Other	B3_10	2014	China: Hubei	Environmental	Fish	VppAsia	NNOU00000000
Other	B3_7	2014	China: Hubei	Environmental	Fish	VppAsia	NNOO00000000

Type	Strain ID	Year	Location	Sample type	Source	Population	Accession Number
Other	B3_8	2014	China: Hubei	Environmental	Fish	VppAsia	NNON00000000
Other	B3_9	2014	China: Hubei	Environmental	Fish	VppAsia	NNOM00000000
Other	B4_10	2014	China: Hubei	Environmental	Fish	VppAsia	NNOK00000000
Other	B4_3	2014	China: Hubei	Environmental	Fish	VppAsia	NNOI00000000
Other	B4_5	2014	China: Hubei	Environmental	Fish	VppX	NNOH00000000
Other	B4_8	2014	China: Hubei	Environmental	Fish	VppAsia	NNOE00000000
Other	B4_9	2014	China: Hubei	Environmental	Fish	VppAsia	NNOD00000000
Other	B5_10	2014	China: Hubei	Environmental	Water	VppAsia	NNOB00000000
Other	B5_2	2014	China: Hubei	Environmental	Fish	VppAsia	NNOA00000000
Other	B6_1	2014	China: Hubei	Environmental	Water	VppAsia	NNNT00000000
Other	B6_10	2014	China: Hubei	Environmental	Water	VppAsia	NNNS00000000
Other	B6_2	2014	China: Hubei	Environmental	Water	VppAsia	NNNR00000000
Other	B6_3	2014	China: Hubei	Environmental	Water	VppAsia	NNNQ00000000
Other	B6_4	2014	China: Hubei	Environmental	Fish	VppAsia	NNNP00000000
Other	B6_6	2014	China: Hubei	Environmental	Fish	VppAsia	NNNN00000000
Other	B6_8	2014	China: Hubei	Environmental	Water	VppAsia	NNNL00000000
Other	B6_9	2014	China: Hubei	Environmental	Fish	VppAsia	NNNK00000000
Other	B7_2	2014	China: Hubei	Environmental	Fish	VppAsia	NNNJ00000000
Other	B7_3	2014	China: Hubei	Environmental	Fish	VppAsia	NNNI00000000
Other	B7_5	2014	China: Hubei	Environmental	Fish	VppAsia	NNNG00000000
Other	B7_6	2014	China: Hubei	Environmental	Fish	VppAsia	NNNF00000000
Other	B8_10	2014	China: Hubei	Environmental	Fish	VppAsia	NNNC00000000
Other	B8_2	2014	China: Hubei	Environmental	Fish	VppAsia	NNNB00000000
Other	B8_4	2014	China: Hubei	Environmental	Fish	VppAsia	NNMZ00000000
Other	B8_7	2014	China: Hubei	Environmental	Fish	VppAsia	NNMW00000000
Other	B8_9	2014	China: Hubei	Environmental	Fish	VppAsia	NNMU00000000
Other	B9_1	2014	China: Hubei	Environmental	Water	VppAsia	NNMT00000000
Other	B9_10	2014	China: Hubei	Environmental	Fish	VppAsia	NNMS00000000
Other	B9_5	2014	China: Hubei	Environmental	Water	VppAsia	NNMO00000000
Other	B9_6	2014	China: Hubei	Environmental	Fish	VppAsia	NNMN00000000
Other	B9_7	2014	China: Hubei	Environmental	Water	VppAsia	NNMM00000000
Other	B9_8	2014	China: Hubei	Environmental	Fish	VppAsia	NNML00000000
Other	C1_2	2014	China: Shandong	Environmental	Fish	VppAsia	NNMI00000000
Other	C1_5	2014	China: Shandong	Environmental	Fish	VppAsia	NNMF00000000
Other	C1_6	2014	China: Shandong	Environmental	Fish	VppAsia	NNME00000000
Other	C1_8	2014	China: Shandong	Environmental	Water	VppAsia	NNMC00000000
Other	C1_9	2014	China: Shandong	Environmental	Water	VppAsia	NNMB00000000
Other	C10_5	2014	China: Zhejiang	Environmental	Fish	VppX	NNLZ00000000
Other	C2_5	2014	China: Shandong	Environmental	Fish	VppAsia	NNLV00000000
Other	C2_6	2014	China: Shandong	Environmental	Fish	VppAsia	NNLU00000000
Other	C2_8	2014	China: Shandong	Environmental	Fish	VppAsia	NNLT00000000
Other	C3_10	2014	China: Shandong	Environmental	Water	VppAsia	NNLS00000000
Other	C3_2	2014	China: Shandong	Environmental	Fish	VppAsia	NNLR00000000
Other	C3_3	2014	China: Shandong	Environmental	Fish	VppAsia	NNLQ00000000
Other	C3_4	2014	China: Shandong	Environmental	Fish	VppX	NNLP00000000
Other	C3_5	2014	China: Shandong	Environmental	Fish	VppX	NNLO00000000
Other	C4_3	2014	China: Shandong	Environmental	Fish	VppAsia	NNLJ00000000
Other	C4_4	2014	China: Shandong	Environmental	Fish	VppAsia	NNLI00000000
Other	C4_5	2014	China: Shandong	Environmental	Fish	VppAsia	NNLH00000000
Other	C4_6	2014	China: Shandong	Environmental	Fish	VppAsia	NNLG00000000
Other	C4_7	2014	China: Shandong	Environmental	Fish	VppAsia	NNLF00000000
Other	C4_8	2014	China: Shandong	Environmental	Fish	VppAsia	NNLE00000000
Other	C5_1	2014	China: Shandong	Environmental	Fish	VppAsia	NNLC00000000
Other	C5_10	2014	China: Shandong	Environmental	Water	VppAsia	NNLB00000000
Other	C5_2	2014	China: Shandong	Environmental	Fish	VppAsia	NNLA00000000
Other	C5_6	2014	China: Shandong	Environmental	Fish	VppX	NNKW00000000
Other	C5_8	2014	China: Shandong	Environmental	Fish	VppAsia	NNKU00000000
Other	C5_9	2014	China: Shandong	Environmental	Fish	VppAsia	NNKT00000000
Other	C6_1	2014	China: Shandong	Environmental	Fish	VppAsia	NNKS00000000
Other	C6_10	2014	China: Shandong	Environmental	Fish	VppAsia	NNKR00000000
Other	C6_2	2014	China: Shandong	Environmental	Fish	VppAsia	NNKQ00000000
Other	C6_4	2014	China: Shandong	Environmental	Fish	VppAsia	NNKP00000000
Other	C6_5	2014	China: Shandong	Environmental	Fish	VppAsia	NNKO00000000
Other	C6_9	2014	China: Shandong	Environmental	Fish	VppAsia	NNKK00000000
Other	C7_1	2014	China: Shandong	Environmental	Water	VppAsia	NNKJ00000000
Other	C7_10	2014	China: Shandong	Environmental	Fish	VppAsia	NNKI00000000
Other	C7_2	2014	China: Shandong	Environmental	Water	VppAsia	NNKH00000000
Other	C7_3	2014	China: Shandong	Environmental	Water	VppAsia	NNKG00000000

Type	Strain ID	Year	Location	Sample type	Source	Population	Accession Number
Other	C7_4	2014	China: Shandong	Environmental	Fish	VppAsia	NNKF00000000
Other	C7_5	2014	China: Shandong	Environmental	Fish	VppAsia	NNKE00000000
Other	C7_6	2014	China: Shandong	Environmental	Fish	VppAsia	NNKD00000000
Other	C7_7	2014	China: Shandong	Environmental	Fish	VppAsia	NNKC00000000
Other	C7_9	2014	China: Shandong	Environmental	Fish	VppAsia	NNKB00000000
Other	C8_3	2014	China: Shandong	Environmental	Fish	VppAsia	NNJZ00000000
Other	C8_5	2014	China: Shandong	Environmental	Water	VppX	NNJX00000000
Other	D1_4	2014	China: Sichuan	Environmental	Fish	VppAsia	NNJR00000000
Other	D1_8	2014	China: Sichuan	Environmental	Fish	VppAsia	NNJP00000000
Other	D2_2	2014	China: Sichuan	Environmental	Fish	VppAsia	NNJO00000000
Other	D2_4	2014	China: Sichuan	Environmental	Fish	VppAsia	NNJN00000000
Other	D2_7	2014	China: Sichuan	Environmental	Fish	VppAsia	NNJM00000000
Other	D2_8	2014	China: Sichuan	Environmental	Fish	VppAsia	NNJL00000000
Other	D2_9	2014	China: Sichuan	Environmental	Fish	VppAsia	NNJK00000000
Other	D3_2	2014	China: Sichuan	Environmental	Fish	VppAsia	NNJJ00000000
Other	D3_5	2014	China: Sichuan	Environmental	Fish	VppAsia	NNJI00000000
Other	D4_3	2014	China: Sichuan	Environmental	Fish	VppAsia	NNJF00000000
Other	D4_5	2014	China: Sichuan	Environmental	Water	VppAsia	NNJD00000000
Other	D4_6	2014	China: Sichuan	Environmental	Fish	VppAsia	NNJC00000000
Other	D4_7	2014	China: Sichuan	Environmental	Water	VppAsia	NNJB00000000
Other	D5_1	2014	China: Sichuan	Environmental	Fish	VppAsia	NNJA00000000
Other	D5_3	2014	China: Sichuan	Environmental	Fish	VppAsia	NNIZ00000000
Other	D5_4	2014	China: Sichuan	Environmental	Fish	VppAsia	NNIY00000000
Other	D5_6	2014	China: Sichuan	Environmental	Fish	VppAsia	NNIX00000000
Other	D5_7	2014	China: Sichuan	Environmental	Water	VppAsia	NNIW00000000
Other	D6_2	2014	China: Sichuan	Environmental	Fish	VppAsia	NNIT00000000
Other	D6_3	2014	China: Sichuan	Environmental	Water	VppAsia	NNIS00000000
Other	D6_4	2014	China: Sichuan	Environmental	Water	VppAsia	NNIR00000000
Other	D6_5	2014	China: Sichuan	Environmental	Fish	VppAsia	NNIQ00000000
Other	D6_6	2014	China: Sichuan	Environmental	Water	VppAsia	NNIP00000000
Other	D6_7	2014	China: Sichuan	Environmental	Fish	VppAsia	NNIO00000000
Other	D6_8	2014	China: Sichuan	Environmental	Fish	VppAsia	NNIN00000000
Other	D7_1	2014	China: Sichuan	Environmental	Fish	VppAsia	NNIM00000000
Other	D7_2	2014	China: Sichuan	Environmental	Water	VppAsia	NNIL00000000
Other	D7_3	2014	China: Sichuan	Environmental	Fish	VppAsia	NNIK00000000
Other	D7_4	2014	China: Sichuan	Environmental	Water	VppAsia	NNIJ00000000
Other	D7_6	2014	China: Sichuan	Environmental	Fish	VppAsia	NNII00000000
Other	D7_7	2014	China: Sichuan	Environmental	Fish	VppAsia	NNIH00000000
Other	D7_8	2014	China: Sichuan	Environmental	Fish	VppAsia	NNIG00000000
Other	D8_3	2014	China: Sichuan	Environmental	Water	VppAsia	NNID00000000
Other	D8_4	2014	China: Sichuan	Environmental	Water	VppAsia	NNIC00000000
Other	D8_5	2014	China: Sichuan	Environmental	Fish	VppAsia	NNIB00000000
Other	D8_7	2014	China: Sichuan	Environmental	Fish	VppAsia	NNHZ00000000
Other	D8_8	2014	China: Sichuan	Environmental	Fish	VppAsia	NNHY00000000
Other	D8_9	2014	China: Sichuan	Environmental	Fish	VppX	NNHX00000000
Other	D9_2	2014	China: Sichuan	Environmental	Fish	VppAsia	NNHW00000000
Other	D9_3	2014	China: Sichuan	Environmental	Fish	VppAsia	NNHV00000000
Other	D9_6	2014	China: Sichuan	Environmental	Fish	VppAsia	NNHT00000000
Other	E1_10	2014	China: Zhejiang	Environmental	Fish	VppAsia	NNHS00000000
Other	E1_5	2014	China: Zhejiang	Environmental	Fish	VppAsia	NNHR00000000
Other	E1_9	2014	China: Zhejiang	Environmental	Fish	VppX	NNHQ00000000
Other	E2_10	2014	China: Zhejiang	Environmental	Fish	VppAsia	NNHN00000000
Other	E2_2	2014	China: Zhejiang	Environmental	Fish	VppAsia	NNHM00000000
Other	E3_1	2014	China: Zhejiang	Environmental	Water	VppAsia	NNHK00000000
Other	E3_10	2014	China: Zhejiang	Environmental	Fish	VppAsia	NNHJ00000000
Other	E4_10	2014	China: Zhejiang	Environmental	Fish	VppAsia	NNHH00000000
Other	E4_3	2014	China: Zhejiang	Environmental	Fish	VppAsia	NNHF00000000
Other	E4_4	2014	China: Zhejiang	Environmental	Fish	VppAsia	NNHE00000000
Other	E4_9	2014	China: Zhejiang	Environmental	Fish	VppAsia	NNHD00000000
Other	E5_8	2014	China: Zhejiang	Environmental	Fish	VppAsia	NNHC00000000
Other	E6_4	2014	China: Zhejiang	Environmental	Water	VppAsia	NNHB00000000
Other	E7_10	2014	China: Zhejiang	Environmental	Fish	VppAsia	NNHA00000000
Other	E7_9	2014	China: Zhejiang	Environmental	Fish	VppAsia	NNGZ00000000
Other	E9_8	2014	China: Zhejiang	Environmental	Shellfish	VppAsia	NNGX00000000
Other	F1_1	2014	China: Guangxi	Environmental	Fish	VppAsia	NNGW00000000
Other	F1_10	2014	China: Guangxi	Environmental	Fish	VppAsia	NNGV00000000
Other	F1_4	2014	China: Guangxi	Environmental	Fish	VppAsia	NNGS00000000
Other	F1_6	2014	China: Guangxi	Environmental	Water	VppAsia	NNGQ00000000

Type	Strain ID	Year	Location	Sample type	Source	Population	Accession Number
Other	F10_1	2014	China: Guangxi	Environmental	Fish	VppAsia	NNGM00000000
Other	F10_10	2014	China: Guangxi	Environmental	Fish	VppAsia	NNGL00000000
Other	F10_2	2014	China: Guangxi	Environmental	Fish	VppAsia	NNGK00000000
Other	F10_4	2014	China: Guangxi	Environmental	Fish	VppAsia	NNGI00000000
Other	F10_5	2014	China: Guangxi	Environmental	Fish	VppAsia	NNGH00000000
Other	F10_7	2014	China: Guangxi	Environmental	Fish	VppAsia	NNGF00000000
Other	F10_8	2014	China: Guangxi	Environmental	Fish	VppAsia	NNGE00000000
Other	F4_10	2014	China: Guangxi	Environmental	Sediment	VppAsia	NNFH00000000
Other	F4_2	2014	China: Guangxi	Environmental	Fish	VppAsia	NNFG00000000
Other	F4_3	2014	China: Guangxi	Environmental	Fish	VppAsia	NNFF00000000
Other	F4_4	2014	China: Guangxi	Environmental	Fish	VppX	NNFE00000000
Other	F4_5	2014	China: Guangxi	Environmental	Fish	VppX	NNFD00000000
Other	F4_8	2014	China: Guangxi	Environmental	Water	VppAsia	NNFA00000000
Other	F4_9	2014	China: Guangxi	Environmental	Sediment	VppAsia	NNEZ00000000
Other	F5_1	2014	China: Guangxi	Environmental	Sediment	VppAsia	NNEY00000000
Other	F5_3	2014	China: Guangxi	Environmental	Fish	VppAsia	NNEW00000000
Other	F5_4	2014	China: Guangxi	Environmental	Fish	VppAsia	NNEV00000000
Other	F5_5	2014	China: Guangxi	Environmental	Fish	VppAsia	NNEU00000000
Other	F5_6	2014	China: Guangxi	Environmental	Fish	VppAsia	NNET00000000
Other	F5_7	2014	China: Guangxi	Environmental	Fish	VppAsia	NNES00000000
Other	F5_8	2014	China: Guangxi	Environmental	Water	VppAsia	NNER00000000
Other	F5_9	2014	China: Guangxi	Environmental	Fish	VppAsia	NNEQ00000000
Other	F6_1	2014	China: Guangxi	Environmental	Water	VppAsia	NNEP00000000
Other	F6_4	2014	China: Guangxi	Environmental	Sediment	VppAsia	NNEM00000000
Other	F6_5	2014	China: Guangxi	Environmental	Fish	VppAsia	NNEL00000000
Other	F6_6	2014	China: Guangxi	Environmental	Fish	VppAsia	NNEK00000000
Other	F6_7	2014	China: Guangxi	Environmental	Fish	VppAsia	NNEJ00000000
Other	F6_8	2014	China: Guangxi	Environmental	Fish	VppAsia	NNEI00000000
Other	F7_10	2014	China: Guangxi	Environmental	Water	VppAsia	NNEG00000000
Other	F7_3	2014	China: Guangxi	Environmental	Water	VppAsia	NNEE00000000
Other	F7_7	2014	China: Guangxi	Environmental	Water	VppAsia	NNEB00000000
Other	F7_8	2014	China: Guangxi	Environmental	Fish	VppAsia	NNEA00000000
Other	F7_9	2014	China: Guangxi	Environmental	Water	VppAsia	NNDZ00000000
Other	F8_1	2014	China: Guangxi	Environmental	Water	VppAsia	NNDY00000000
Other	F8_3	2014	China: Guangxi	Environmental	Water	VppX	NNDV00000000
Other	F8_5	2014	China: Guangxi	Environmental	Fish	VppAsia	NNDT00000000
Other	F8_6	2014	China: Guangxi	Environmental	Fish	VppAsia	NNDS00000000
Other	F8_7	2014	China: Guangxi	Environmental	Fish	VppAsia	NNDR00000000
Other	F8_8	2014	China: Guangxi	Environmental	Fish	VppAsia	NNDQ00000000
Other	F8_9	2014	China: Guangxi	Environmental	Fish	VppAsia	NNDP00000000
Other	F9_1	2014	China: Guangxi	Environmental	Fish	VppAsia	NNDO00000000
Other	F9_10	2014	China: Guangxi	Environmental	Fish	VppAsia	NNDN00000000
Other	F9_2	2014	China: Guangxi	Environmental	Fish	VppAsia	NNDM00000000
Other	F9_3	2014	China: Guangxi	Environmental	Fish	VppAsia	NNDL00000000
Other	F9_5	2014	China: Guangxi	Environmental	Fish	VppAsia	NNDJ00000000
Other	F9_6	2014	China: Guangxi	Environmental	Fish	VppAsia	NNDI00000000
Other	F9_7	2014	China: Guangxi	Environmental	Fish	VppAsia	NNDH00000000
Other	F9_8	2014	China: Guangxi	Environmental	Fish	VppAsia	NNDG00000000
Other	F9_9	2014	China: Guangxi	Environmental	Fish	VppAsia	NNDF00000000
Other	G1_2	2014	China: Guangxi	Environmental	Fish	VppAsia	NNDC00000000
Other	G2_3	2014	China: Guangxi	Environmental	Fish	VppAsia	NNCS00000000
Other	G2_5	2014	China: Guangxi	Environmental	Fish	VppAsia	NNCR00000000
Other	G2_6	2014	China: Guangxi	Environmental	Water	VppAsia	NNCQ00000000
Other	S010	NA	NA	NA	NA	VppAsia	NNCO00000000
Other	S107	NA	NA	NA	NA	VppAsia	NNCL00000000
Other	S149	NA	NA	NA	NA	VppAsia	NNCK00000000
Other	S178	NA	NA	NA	NA	VppAsia	NNCG00000000

Supplementary Table 4 The PCR primers used to identify *V. parahaemolyticus* strain

Primer	Sequence (3' to 5')	Target gene	Annealing temperature (°C)	Cycling number	Amplicon （bp)	Reference
toxR- F	TGTACTGTTGAACGCCTAA	*toxR*	55	30	503	(1)
toxR -R	CACGTTCTCATACGAGTG					
tdh- F	GTAAAGGTCTCTGACTTTTGGAC	*tdh*	60	30	270	(2)
tdh -R	TGGAATAGAACCTTCATCTTCACC					
trh -F	TTGGCTTCGATATTTTCAGTATCT	*trh*	60	30	500	(2)
trh -R	AACAAACATATGCCCATTTCCG					
tlh -R	AAAGCGGATTATGCAGAAGCACTG	*tlh*	60	30	450	(2)
tlh -F	GCTACTTTCTAGCATTTTCTCTGC					
Intl1-F	TCATGGCTTGTTATGACTGT	Intl1	52	30	403	(3, 4)
Intl1-R	GTAGGGCTTATTATGCACGC					
Intl2-F	GATGCCATCGCAAGTACGAG	Intl2	52	30	717	(3, 4)
Intl2-R	CGGGATCCCGGACGGCATGCACGATTTGTA					

References

1. Park JY, Jeon S, Kim JY, Park M, Kin S. 2013. Multiplex Real-time Polymerase Chain Reaction Assays for Simultaneous Detection of *Vibrio cholerae* , *Vibrio parahaemolyticus* , and *Vibrio vulnificus* . Osong public health and research perspectives 4: 133-139.

2. Bej AK, Patterson DP, Brasher CW, Vickey MC, Jones DD, Kaysner CA. 1999. Detection of total and hemolysin-producing *Vibrio parahaemolyticus* in shellfish using multiplex PCR amplification of *tl* , *tdh* and *trh* . Journal microbiological methods 36: 215-225.

3. Su J, Shi L, Yang L, Xiao Z, Li X, Yamasaki S. 2006. Analysis of integrons in clinical isolates of *Escherichia coli* in China during the last six years. FEMS microbiology letters 254: 75-80.

4. Blahna MT, Zalewski CA, Reuer J, Kahlmeter G, Foxman B, Marrs CF. 2006. The role of horizontal gene transfer in the spread of trimethoprim-sulfamethoxazole resistance among uropathogenic *Escherichia coli* in Europe and Canada. Journal of antimicrobial chemotherapy 57: 666-672.

Supplementary Table 5 The information of the sequences similar to plasmid pCol3M

Description	Scientific Name	Max Score	Total Score	Query Cover	E value	Per. Ident	Acc. Len	Accession
Proteus mirabilis plasmid pM510, complete sequence	*Proteus mirabilis*	4865	5191	100%	0	100.00%	2683	KJ190020.1
Proteus mirabilis plasmid pRS12-11, complete sequence	*Proteus mirabilis*	4841	5191	100%	0	100.00%	2683	KF364953.1
Proteus vulgaris strain Q5169 plasmid pEAD1-1, complete sequence	*Proteus vulgaris*	4619	5191	100%	0	100.00%	2683	KF498970.1
Proteus mirabilis strain 1035 plasmid pPM1035, complete sequence	*Proteus mirabilis*	4619	5191	100%	0	100.00%	2683	CP072780.1
Proteus mirabilis strain 1035 plasmid pPM1035, complete sequence	*Proteus mirabilis*	4619	5191	100%	0	100.00%	2683	MW248467.1
Proteus mirabilis strain SCSZC10 plasmid pSCSZC10-qnrD1, complete sequence	*Proteus mirabilis*	4547	5191	100%	0	100.00%	2683	MT349898.1
Proteus mirabilis strain SCRNC2 plasmid pSCRNC2-qnrD1, complete sequence	*Proteus mirabilis*	4547	5191	100%	0	100.00%	2683	MT349897.1
Proteus mirabilis strain HERJC5 plasmid pHERJC5-qnrD1, complete sequence	*Proteus mirabilis*	4547	5191	100%	0	100.00%	2683	MT349895.1
Proteus mirabilis strain HBSZC6 plasmid pHBSZC6-qnrD1, complete sequence	*Proteus mirabilis*	4547	5191	100%	0	100.00%	2683	MT349892.1
Proteus mirabilis strain HBSZC3 plasmid pHBSZC3-qnrD1, complete sequence	*Proteus mirabilis*	4547	5191	100%	0	100.00%	2683	MT349891.1
Proteus mirabilis strain HBDW1 plasmid pHBDW1-qnrD1, complete sequence	*Proteus mirabilis*	4547	5191	100%	0	100.00%	2683	MT349890.1
Providencia stuartii strain MF1 plasmid pMF1A, complete sequence	*Providencia stuartii*	4418	5191	100%	0	100.00%	2683	CP048622.1
Proteus sp. ZN5 plasmid pZN5-qnrD, complete sequence	*Proteus sp. ZN5*	4161	5191	100%	0	100.00%	2683	CP047641.1
Proteus terrae subsp. cibarius strain ZF2 plasmid pZF2-qnrD, complete sequence	*Proteus terrae subsp. cibarius*	4037	5191	100%	0	100.00%	2683	CP045011.1
Proteus mirabilis strain ZA25 plasmid pZA25-qnrD, complete sequence	*Proteus mirabilis*	3989	5191	100%	0	100.00%	2683	CP047354.1
Proteus terrae subsp. cibarius strain ZF1 plasmid pZF1-qnrD, complete sequence	*Proteus terrae subsp. cibarius*	3729	5191	100%	0	100.00%	2683	CP047343.1
Salmonella enterica subsp. enterica serovar Heidelberg strain 69 plasmid, partial sequence	*Salmonella enterica subsp. enterica serovar Heidelberg*	3441	5191	100%	0	100.00%	2683	MK191843.1
Morganella morganii strain MP63 plasmid pMP63B, complete sequence	*Morganella morganii*	3411	5191	100%	0	100.00%	2683	CP048808.1
Proteus sp. NMG38-2 plasmid p2_NMG38-2, complete sequence	*Proteus sp. NMG38-2*	3182	5191	100%	0	100.00%	2683	CP085484.1
Proteus mirabilis strain HBRJC9 plasmid pHBRJC9-qnrD1, complete sequence	*Proteus mirabilis*	3040	5762	100%	0	100.00%	4538	MT349901.1
Proteus vulgaris plasmid pRS12-78, complete sequence	*Proteus vulgaris*	3040	5229	100%	0	100.00%	4286	KF364954.1
Salmonella enterica subsp. enterica serovar Braenderup strain 95.50 plasmid KVHS-001 transposase (insB) and plasmid mediated quinolone resistance protein (qnrD1) genes, complete cds	*Salmonella enterica subsp. enterica serovar Braenderup*	2939	5207	100%	0	100.00%	6887	KJ685894.1
Salmonella enterica subsp. enterica serovar Typhimurium strain 14620 plasmid KVHS-002 transposase (insB) and plasmid mediated quinolone resistance protein (qnrD1) genes, complete cds	*Salmonella enterica subsp. enterica serovar Typhimurium*	2939	5207	100%	0	100.00%	8127	KJ685893.1
Salmonella enterica subsp. enterica serovar Montevideo strain 29722/48 plasmid KVHS-003 transposase (insB) and plasmid mediated quinolone resistance protein (qnrD1) genes, complete cds	*Salmonella enterica subsp. enterica serovar Montevideo*	2939	5207	100%	0	100.00%	9384	KJ685891.1
Proteus mirabilis strain CGP248 plasmid pCGP248, complete sequence	*Proteus mirabilis*	2915	5191	100%	0	100.00%	2683	JQ776503.1
Morganella morganii strain Vr831 plasmid p831 pentapeptide repeat protein (qnrD) and hypothetical protein genes, complete cds	*Morganella morganii*	2915	5163	100%	0	100.00%	2684	JN183061.1
Proteus mirabilis strain T80 plasmid pT80 pentapeptide repeat protein (qnrD) and hypothetical protein genes, complete cds	*Proteus mirabilis*	2915	5167	100%	0	100.00%	2687	JN183060.1
Proteus terrae subsp. cibarius strain ZN2 plasmid pZN2-qnrD, complete sequence	*Proteus terrae subsp. cibarius*	2876	5191	100%	0	100.00%	2683	CP047351.1
Proteus mirabilis strain PmZXF plasmid pPmZXF, complete sequence	*Proteus mirabilis*	2846	5185	100%	0	100.00%	2683	KP313759.1
Proteus mirabilis strain PmBR607 plasmid unnamed2, complete sequence	*Proteus mirabilis*	2769	5191	100%	0	100.00%	2683	CP049755.1
Proteus terrae subsp. cibarius strain SDQ8C180-2T plasmid pSDQ8C180-2, complete sequence	*Proteus terrae subsp. cibarius*	2567	5569	100%	0	100.00%	5202	CP073358.1
Proteus mirabilis strain CRE14IB plasmid pIB_COL3M, complete sequence	*Proteus mirabilis*	2819	5139	99%	0	100.00%	2655	CP045539.1
Moellerella wisconsensis strain W51 plasmid pW51-c, complete sequence	*Moellerella wisconsensis*	3515	5082	97%	0	100.00%	2624	CP093248.1
Providencia alcalifaciens strain BT169 plasmid pBT169, complete sequence	*Providencia alcalifaciens*	4835	5185	100%	0	99.96%	2683	MH085194.1
Proteus mirabilis plasmid pRS12-104, complete sequence	*Proteus mirabilis*	4835	5185	100%	0	99.96%	2683	KF364955.1
Providencia rettgeri strain YPR25 plasmid pYPR25-3, complete sequence	*Providencia rettgeri*	4835	5185	100%	0	99.96%	2683	CP060729.1
Proteus mirabilis strain C1042 plasmid p1042, complete sequence	*Proteus mirabilis*	4833	5183	100%	0	99.96%	2682	KP330456.1
Proteus mirabilis strain 39224 plasmid p39224, complete sequence	*Proteus mirabilis*	4614	5185	100%	0	99.96%	2683	MF062094.1
Proteus vulgaris strain 36852 plasmid p36852, complete sequence	*Proteus vulgaris*	4614	5185	100%	0	99.96%	2683	MF062093.1
Proteus mirabilis strain SCSZC22 plasmid pSCSZC22-qnrD1, complete sequence	*Proteus mirabilis*	4542	5185	100%	0	99.96%	2683	MT349899.1
Citrobacter freundii strain RHB14-C12 plasmid pRHB14-C12_2, complete sequence	*Citrobacter freundii*	4137	5185	100%	0	99.96%	2683	CP057825.1
Proteus mirabilis plasmid pRS12-189, complete sequence	*Proteus mirabilis*	4785	5135	99%	0	99.96%	2656	KF364956.1
Moellerella wisconsensis strain W17-3 plasmid pW17-3-b, complete sequence	*Moellerella wisconsensis*	3530	5185	100%	0	99.95%	2683	CP093262.1
Morganella morganii plasmid pM60, complete sequence	*Morganella morganii*	3343	5185	100%	0	99.94%	2683	KF813021.1
Salmonella enterica subsp. enterica serovar Hadar strain 139.67 plasmid KVHS-004 plasmid mediated quinolone resistance protein (qnrD1) and regulator protein (rop) genes, complete cds	*Salmonella enterica subsp. enterica serovar Hadar*	2955	5198	100%	0	99.94%	6227	KJ685892.1
Proteus vulgaris strain MB18 plasmid pMB18, complete sequence	*Proteus vulgaris*	2907	5617	100%	0	99.94%	5201	KM577619.1
Providencia rettgeri strain AB213 plasmid pAB213, complete sequence	*Providencia rettgeri*	4830	5180	100%	0	99.92%	2683	MH085193.1
Proteus mirabilis strain HBNNC22 plasmid pHBNNC22-qnrD1, complete sequence	*Proteus mirabilis*	4536	5180	100%	0	99.92%	2683	MT349896.1
Proteus vulgaris strain ZN3 plasmid pZN3-qnrD, complete sequence	*Proteus vulgaris*	3615	5180	100%	0	99.90%	2683	CP047348.1
Providencia rettgeri plasmid pDIJ09-518a, complete sequence	*Providencia rettgeri*	4824	5174	100%	0	99.89%	2683	HQ834472.1
Proteus mirabilis strain HBSZC23 plasmid pHBSZC23-qnrD1, complete sequence	*Proteus mirabilis*	4530	5174	100%	0	99.88%	2683	MT349893.1
Proteus sp. 3M plasmid p3M-2B, complete sequence	*Proteus sp. 3M*	3044	6011	100%	0	99.88%	5903	JX514066.1
Providencia rettgeri plasmid pGHS09-09a, complete sequence	*Providencia rettgeri*	4813	5163	100%	0	99.81%	2683	HQ834473.1
Ochrobactrum anthropi plasmid pOA8912 complete sequence	*Brucella anthropi*	2898	5152	100%	0	99.81%	2683	HF679278.1
Morganella morganii strain CGH69 plasmid pCGH69, complete sequence	*Morganella morganii*	2898	5152	100%	0	99.81%	2683	JQ776510.1
Proteus mirabilis strain HBSZC25 plasmid pHBSZC25-qnrD1, complete sequence	*Proteus mirabilis*	4519	5152	100%	0	99.80%	2683	MT349894.1
Proteus mirabilis strain HBDJC1 plasmid pHBDJC1-qnrD1, complete sequence	*Proteus mirabilis*	4514	5147	100%	0	99.76%	2683	MT349889.1
Proteus penneri strain 22499 plasmid p22499, complete sequence	*Proteus penneri*	4547	5119	100%	0	99.48%	2683	MF062092.1
Proteus mirabilis strain RGF134-1 plasmid pRGF134-1-5kb, complete sequence	*Proteus mirabilis*	5107	8439	100%	0	99.47%	5947	CP066835.1
Proteus mirabilis strain Q1084 plasmid pEAD1-2, complete sequence	*Proteus mirabilis*	4266	4790	99%	0	97.70%	2669	KF498971.1
Proteus mirabilis strain 39190 plasmid p39190, complete sequence	*Proteus mirabilis*	4255	4796	99%	0	97.62%	2669	MF062091.1
Serratia marcescens Sm3 plasmid pSm3qnr DNA, complete sequence	*Serratia marcescens*	2708	5174	100%	0	97.60%	5204	LC543863.1
Proteus mirabilis strain 36854 plasmid p36854, complete sequence	*Proteus mirabilis*	4028	4513	99%	0	95.98%	2669	MF062090.1
Proteus mirabilis strain 33184 plasmid p33184, complete sequence	*Proteus mirabilis*	3984	4491	99%	0	95.74%	2657	MF062089.1
Proteus mirabilis plasmid pLRB12-304, complete sequence	*Proteus mirabilis*	4191	4508	100%	0	95.69%	2658	KF364957.1
Morganella morganii strain SE10MM plasmid pSE10MM, complete sequence	*Morganella morganii*	4098	4373	100%	0	94.79%	2662	KU160530.1

Supplementary Table 6 The genome sequence quality data of 124 *V. parahaemolyticus* strains in this study

Isolate name	AvgQuality	N50	NrContigs	length of assembled genome	NrConsensus	GC%
SH2-1	29746.1	451185	183	5443542	2252	45.1
SH2-2	80775.7	379247	65	5250422	2252	45.3
SH4-3	29037.7	484917	189	5488132	2252	45
SH5-1	72196.1	833232	69	4981528	2253	45.4
SH7-1	76031.1	752950	66	5018050	2258	45.4
SH7-2	27484.0	542492	200	5496810	2262	44.6
SH8-1	59322.7	389147	100	5932271	2258	44.1
SH8-2	84711.3	520564	69	5845083	2253	44.1
SH10-3	21898.6	321425	241	5277558	2266	45.1
SH10-4	60561.3	550459	83	5026585	2250	45.3
SH11-1	63357.8	386680	78	4941907	2252	45.4
SH13-1	16315.8	559111	343	5596333	2257	45.0
SH14-1	80817.7	555389	62	5010699	2260	45.4
SH14-2	47187.8	494874	106	5001905	2257	45.4
SH15-1	81248.6	545398	64	5199912	2251	45.2
SH15-2	66706.4	402301	78	5203096	2258	45.3
SH16-1	83781.8	404865	63	5278254	2252	45.2
SH16-2	75635.4	478301	70	5294478	2252	45.2
FT2-1	71957.2	891607	71	5108963	2253	45.3
FT2-2	91308.8	572348	54	4930677	2254	45.4
FT3-1	43356.3	1018916	116	5029332	2254	45.3
FT3-2	23093.5	612059	246	5681001	2255	44.6
FT8-1	135742.4	747487	37	5022468	2257	45.3
FT8-2	81675.9	478234	61	4982229	2256	45.3
1-1	90166.6	499153	55	4959165	2254	45.4
1-2	41955.5	361812	125	5244434	2250	45.2
2-1	65146.0	342903	78	5081385	2250	45.3
3-1	64266.5	530109	78	5012784	2260	45.4
3-2	94313.4	531584	53	4998611	2249	45.4
4-1	71619.7	405914	71	5084998	2258	45.4
4-2	71625.1	405914	71	5085385	2258	45.4
5-1	90736.1	449153	55	4990488	2267	45.4
5-2	84998.8	505921	59	5014932	2260	45.3
6-1	42628.5	361812	123	5243304	2250	45.2
6-2	97422.5	432637	51	4968550	2265	45.4
7-1	88349.5	731224	57	5035924	2253	45.4
7-2	91346.7	433068	55	5024068	2259	45.4
8-1	68906.5	540610	75	5167984	2259	45.2
8-2	81029.5	464786	62	5023828	2253	45.4
9-1	70822.8	487291	71	5028417	2256	45.4
9-2	72868.0	549649	69	5027890	2256	45.4
10-1	80366.2	452560	62	4982707	2258	45.4
10-2	83040.7	548082	60	4982441	2258	45.4
11-1	84228.6	376134	60	5053718	2252	45.3
11-2	42078.8	338577	122	5133619	2256	45.4
12-1	91565.7	450431	54	4944549	2257	45.4
13-1	42773.5	379904	120	5132819	2256	45.4
13-2	39151.7	213796	128	5011422	2249	45.3
14-1	42425.6	401172	121	5133502	2256	45.4
14-2	49084.6	448655	103	5055710	2262	45.3
15-1	58464.3	211237	89	5203323	2254	45.1
15-2	62471.5	441049	82	5122666	2251	45.3
16-1	54476.1	530116	93	5066274	2261	45.3
16-2	64237.4	457884	78	5010519	2259	45.4
17-1	81092.0	412630	63	5108798	2257	45.2
18-1	53904.4	530116	94	5067009	2261	45.3
18-2	56377.2	466729	89	5017575	2257	45.3
20-1	73531.2	737378	67	4926591	2245	45.5
20-2	71902.0	534844	70	5033137	2253	45.4
21-1	72313.7	570723	69	4989643	2252	45.4
21-2	68314.2	547693	74	5055249	2252	45.4
23-1	58913.2	402551	85	5007625	2254	45.4
23-2	70464.2	455331	71	5002957	2258	45.4
24-1	61394.0	290897	83	5095701	2253	45.3
24-2	92794.8	704315	55	5103712	2255	45.3
25-1	54884.7	461148	91	4994511	2251	45.3
25-2	72507.5	461957	70	5075527	2262	45.3
26-1	55681.7	530116	91	5067031	2261	45.3
26-2	72137.7	317443	74	5338187	2228	45.2
27-1	63550.0	289275	82	5211101	2249	45.2
27-2	73062.0	466851	69	5041276	2252	45.3
28-1	86711.8	358370	58	5029283	2232	45.3
28-2	117236.3	734965	42	4923926	2254	45.4
29-1	116066.9	452091	43	4990876	2252	45.3
29-2	39225.7	232056	131	5138570	2254	45.3
31-1	97513.6	789525	51	4973194	2260	45.4
31-2	100462.1	460052	49	4922641	2251	45.5
32-1	85201.6	784511	59	5026895	2260	45.4
32-2	89763.5	784511	56	5026756	2260	45.4
33-1	83151.6	262526	65	5404853	2227	45.1

Isolate name	AvgQuality	N50	NrContigs	length of assembled genome	NrConsensus	GC%
33-2	90066.3	308794	60	5403979	2227	45.1
34-1	69490.5	442412	72	5003319	2258	45.4
34-2	86457.6	541740	58	5014542	2261	45.4
35-1	85357.3	537969	59	5036079	2250	45.4
35-2	88347.0	537969	57	5035779	2250	45.4
36-1	66247.4	455156	77	5101047	2259	45.3
36-2	54710.9	515695	92	5033407	2256	45.4
37-1	71428.4	451741	70	4999987	2262	45.3
37-2	75001.8	455156	68	5100123	2259	45.3
38-1	68738.5	373166	74	5086646	2266	45.3
38-2	62364.2	375609	81	5051499	2254	45.3
39-1	33443.1	383508	153	5116787	2253	45.2
40-1	31802.2	381501	162	5151953	2259	45.2
40-2	68940.8	455156	74	5101620	2259	45.3
41-1	54119.8	515695	93	5033137	2256	45.4
41-2	68026.4	455156	75	5101978	2259	45.3
42-1	47556.0	262928	112	5326277	2247	45.1
42-2	47657.2	393098	105	5004008	2247	45.4
WS1-1	59158.6	731324	85	5028478	2247	45.3
F7-1	81988.3	459632	64	5247250	2252	45.2
F7-2	48535.1	346756	104	5047646	2251	45.2
F14-1	65529.7	338919	83	5438963	2225	45.1
F14-2	66327.5	338919	82	5438856	2225	45.1
WF2-1	64915.1	401724	78	5063381	2265	45.3
WF2-2	84673.0	340003	63	5334397	2251	45.2
Z8-1	58736.0	521491	86	5051300	2260	45.4
Z11-1	81887.2	524767	60	4913234	2254	45.4
Z11-2	75662.4	460977	68	5145040	2251	45.3
Z13-1	65060.7	378283	80	5204859	2265	45.2
Z13-2	65894.3	367553	79	5205647	2265	45.2
Z14-1	28014.5	91656	192	5378788	2230	45.2
Z14-2	65891.3	378283	79	5205413	2265	45.2
Z19-1	75411.7	378283	69	5203405	2265	45.2
L2-1	48039.2	397951	110	5284316	2258	45.2
L14-1	50977.4	445523	99	5046763	2251	45.2
L14-2	22078.9	100947	232	5122301	2240	45.2
L15-1	38272.4	452378	139	5319870	2258	45.1
L20-1	38271.1	452378	139	5319689	2258	45.1
L21-1	77814.9	373745	64	4980153	2258	45.4
L23-1	41321.1	289868	122	5041175	2251	45.2
L23-2	46244.9	232733	109	5040692	2251	45.2
L26-1	37926.7	264114	134	5082179	2256	45.2
L28-1	36284.0	264753	139	5043478	2250	45.2
L29-1	42717.0	289886	118	5040607	2251	45.2
